# Chemoreversal Agents from Taiwanofungus Genus and Their More Potent Methyl Derivatives Targeting Signal Transducer and Activator of Transcription 3 (STAT3) Phosphorylation

**DOI:** 10.3390/ph14090916

**Published:** 2021-09-10

**Authors:** Ko-Hua Yu, Chin-Chuan Hung, Tian-Shung Wu, Chin-Fu Chen, I-Ting Wu, Ping-Chung Kuo, Sio-Hong Lam, Hsin-Yi Hung

**Affiliations:** 1 School of Pharmacy, College of Medicine, National Cheng Kung University, Tainan 701, Taiwan; s68071061@gs.ncku.edu.tw (K.-H.Y.); tswu@mail.ncku.edu.tw (T.-S.W.); z10502016@email.ncku.edu.tw (P.-C.K.); shlam@mail.ncku.edu.tw (S.-H.L.); 2 Department of Pharmacy, College of Pharmacy, China Medical University, Taichung 406, Taiwan; cchung@mail.cmu.edu.tw (C.-C.H.); u105015202@cmu.edu.tw (I.-T.W.); 3 Department of Pharmacy, China Medical University Hospital, Taichung 404, Taiwan; 4 Department of Healthcare Administration, Asia University, Taichung 500, Taiwan; 5 Department of Pharmacy, College of Pharmacy and Health Care, Tajen University, Pingtung 907, Taiwan; 6 Department of Life Sciences, National Cheng Kung University, Tainan 701, Taiwan; chinfu9999@gmail.com

**Keywords:** multidrug resistance, signal transducer and activator of transcription 3 (STAT3), zhankuic acid

## Abstract

Multidrug resistance (MDR), for which the mechanisms are not yet fully clear, is one of the major obstacles to cancer treatment. In recent years, signal transducer and activator of transcription 3 (STAT3) were found to be one of the important MDR mechanism pathways. Based on the previous research, zhankuic acid A, B, and C were found to have collateral sensitivity effects on MDR cancer cells, and MDR inhibitory activity of zhankuic acid methyl ester was found to be better than that of its acid. Therefore, we executed a systematic examination of the structure–activity relationship of zhankuic acid methyl ester derivatives to collateral sensitivity in MDR cancer cells. The results showed that compound **12** is the best in terms of chemoreversal activity, where the reversal fold was 692, and the IC_50_ value of paclitaxel combined with 10 μM compound **12** treatment was 1.69 nM in MDR KBvin cells. Among all the derivatives, methyl ester compounds were found to be better than their acids, and a detailed discussion of the structure–activity relationships of all of the derivatives is provided in this work. In addition, compounds **8**, **12,** and **26** were shown to influence the activation of STAT3 in KBvin cells, accounting for part of their chemoreversal effects. Our results may provide a new combined therapy with paclitaxel to treat multidrug-resistant cancers and provide a new therapy option for patients.

## 1. Introduction

Although various advanced cancer therapies have emerged, chemotherapy remains an effective treatment for cancer patients. However, multidrug resistance (MDR), which occurs during or after treatment in a short period, is one of the obstacles accounting for cancer treatment failure [1,2,3]. There are a number of reasons for the formation of MDR, including irregular metabolism, distribution, and absorption to target cells. As cancer cells develop multidrug resistance, the anti-cancer efficacy of chemotherapeutic drugs decreases, which subsequently leads to cancer metastasis and recurrence [4]. Mechanisms of multidrug resistance have been studied intensively, including drug efflux, growth factors, genetic factors, and increased DNA repair ability [4,5,6,7]. For example, ATP-binding cassette (ABC) proteins, such as P-glycoprotein (P-gp), play important roles in multidrug resistance and have drawn much attention due to their potential for therapeutic usage [8,9].

Unfortunately, P-gp inhibitors have failed to achieve clinical use due to drug toxicity, adverse drug interactions, and pharmacokinetic issues [10]. Recently, a phenomenon called collateral sensitivity, defined as sensitizing cancer cells to chemotherapeutic agents by another agent was proven to have therapeutic potential clinically [11]. Several molecular collateral sensitivity mechanisms were found, including elevation of ATP hydrolysis, alterations of drug target proteins, and increased generation of reactive oxygen species [12]. In addition, as identified by our group [13], danazol has demonstrated a collateral sensitivity effect via reduction of phosphorylation of signal transducer and activator of transcription 3 (STAT3) and reduction of STAT3-regulated down-stream signals in MDR cells [14].

In addition, STAT3 can be upregulated by Janus-activated kinase 2 (JAK 2), toll-like receptor 4 (TLR 4), human epidermal growth factor receptor (EGFR), IL-6-type family, and several G protein-coupled receptors (GPCRs), which significantly associates development of resistance in various cancers [15,16,17]. Therefore, inhibition of STAT3 activation can be an effective method to reduce resistant cancer cell growth. For instance, inhibition of the JAK2/STAT3 pathway has been shown to reverse paclitaxel resistance in human ovarian tumors [18]. By silencing STAT3, cancer resistance to doxorubicin, cisplatin, and paclitaxel can be re-sensitized [19]. Moreover, through induction of the IL-6/STAT3 pathway, the estrogen receptor and DNA repair were downregulated, and inhibition of STAT3 and PARP (poly ADP-ribose polymerase) induced cell death in the palbociclib-resistant cells [20]. Moreover, overexpression of PAX3 (paired box homeotic gene 3) or activation of STAT3 led to vemurafenib, a selective inhibitor of Braf, resistance in melanoma cells, and silencing of PAX3 and STAT3 reduced growth of vemurafenib resistance melanoma cells [21,22].

**Figure 1:**
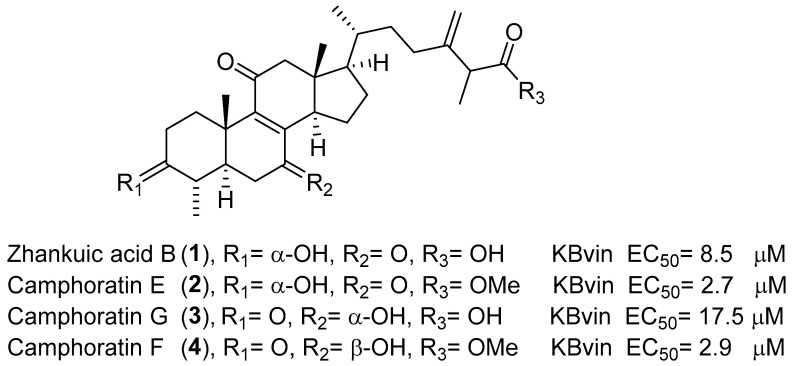
Cytotoxicity of compound **1**–**4** isolated from *T. camphoratus* on KBvin cell lines.

In 2017, the *n*-hexane extract of *Taiwanofungus camphoratus* exhibited inhibitory activity on STAT3 pathways in EGFR wild-type NSCLC (non-small cell lung cancer) cells [23]. However, the active principals were not identified in this study. Our group has studied *Taiwanofungus camphoratus* and its related species, *T. salmoneus* for a while [24,25,26,27]. Our previous studies showed that zhankuic acid A, B, and C, major triterpenoids in *Taiwanofungus camphoratus*, have collateral sensitivity effects on the MDR cancer cell line (KBvin) and P-gp inhibitory effects on the P-gp over-expressed cell line (ABCB1/Flp-In^TM^-293) [27,28,29]. In addition, zhankuic acid A can bind to the TLR4 receptor and block an LPS-induced inflammation cascade [30]. Moreover, zhankuic acid A can also bind to JAK2 and block downstream signals, including STAT3 phosphorylation [31]. In MDR research, we found an interesting phenomenon indicating that the MDR inhibitory ability of zhankuic acid methyl ester was better than that of its acid, zhankuic acid, in KBvin cells (Figure 1) [25]. For example, camphoratin E (**2**) (EC_50_ = 2.7 µM), the methyl ester of zhankuic acid B (**1**), was shown to be more potent than its acid form (EC_50_ = 8.5 µM). The same trend was also observed in the camphoratin G (**3**) and camphoratin F (**4**) pair [25]. This phenomenon aroused our interest. Therefore, we planned to conduct a systematic examination of the structure–activity relationships among zhankuic acid type compounds and their methyl ester derivatives on chemoreversal activity in MDR cancer cell lines. Furthermore, the action mechanism of STAT3 phosphorylation of the most active compound pairs was studied.

## 2. Results and Discussion

### 2.1. Chemistry

**Figure 2:**
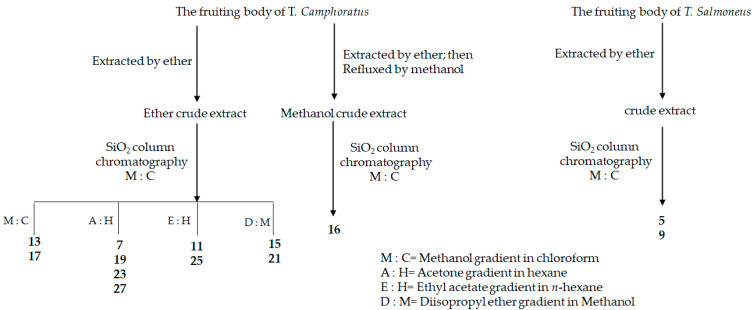
Isolation flow chart for zhankuic acid-type compounds.

Following previous studies, a variety of zhankuic acid-type compounds were selected and gathered from our group based on their structures and available quantity. These compounds were isolated from the fruiting body of *T. camphoratus* and *T. salmoneus* [25,27,32,33]. The dried plant materials were extracted by ether or methanol, partitioned, and then undergone repeated silica gel chromatography with different eluents to obtain zhankuic acid type compounds. The isolation process is summarized in Figure 2 and the names and references are shown in Table 1 [25,27,32,33].

**Scheme 1:**
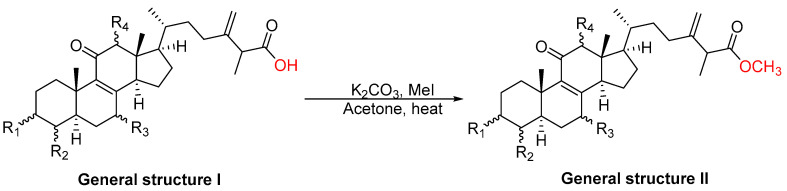
Synthetic scheme of methyl ester derivatives.

The collected zhankuic acid type compounds (**general structure I**) were reacted with potassium carbonate and iodomethane to obtain their methyl esters (**general structure II**) with good yield (80–92%) (Scheme 1). The multidrug-resistant reversal ability of the twelve acid natural products (**5**, **7**, **9**, **11**, **13**, **15**, **17**, **19**, **21**, **23**, **25** and **27**) and their methyl esters (**6**, **8**, **10**, **12**, **14**, **16**, **18**, **20**, **22**, **24**, **26** and **28**) were all evaluated on MDR strain KBvin and non-MDR HeLa S3 cells.

### 2.2. Cytotoxic Evaluation of Compounds **5**–**28** on Hela S3 and KBvin Cells

Firstly, all derivatives were tested for cytotoxicity in HeLa S3 and KBvin cells (Table 2). Generally, the methyl ester derivatives were more cytotoxic than their acid compounds. The most extreme example (compound pair **5** and **6** (IC_50_ value of >40 and 19.83 μM in KBvin, respectively) exhibited a twofold difference in IC_50_ values. Besides the methyl ester in the R_5_ position, various substituents in the R_1_ position had no significant effects on the activity, as evidenced by **12** with the carbonyl group and **28** with α-hydroxy substitution (IC_50_ values of 22.29 μM and 30.98 μM in KBvin, respectively). More evidence was observed on **18** with an α-*O*-formate substituent and compound **10** with a carbonyl substituent (IC_50_ values of 20.95 μM and 21.77 μM in KBvin, respectively). In the R_3_ position, the hydroxy group can improve cytotoxicity regardless of its α or β position, such as compounds **6** and **26** (IC_50_ values of 19.83 μM and 27.32 μM in KBvin, respectively). In addition, carbonyl and β-methyl of R_3_ substituents are also beneficial, such as **8**, **18** and **22** (IC_50_ values of 24.44, 20.95 and 14.48 μM in KBvin, respectively). On the contrary, hydrogen in the R_3_ position, such as **24** and **28,** results in a deterioration of the cytotoxic effect (IC_50_ values of >40 and 30.98 μM in KBvin, respectively). Based on the results indicating that the lowest IC_50_ values for the KBvin and HeLa S3 cells were 14 μM and 21 μM, two sets of concentrations below IC_50_ for both cells, which ruled out effects due to cytotoxicity, were selected for further collateral sensitivity evaluation.

### 2.3. Collateral Sensitivity Evaluation of the Effects of Zhankuic Acid Type Compounds and Their Methyl Esters on Paclitaxel Cytotoxicity

**Figure 3:**
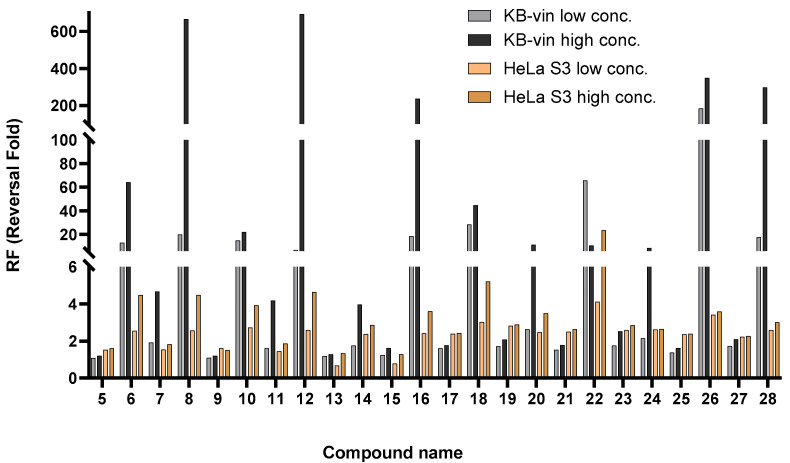
Reversal fold values of zhankuic acid type compounds and their methyl esters in KBvin and HeLa S3 cells. For R_5_ acid-substituted compounds (odd compound number), the low concentration (conc.) was 10 μM, and the high conc. was 20 μM. For the R_5_ ester-substituted compounds (even compound number), the low conc. was 5 μM, and the high conc. was 10 μM.

Collateral sensitivity of all the compounds was determined based on the anti-proliferative effects of both HeLa and KBvin cells under co-treatment with paclitaxel and the compounds. Reversal fold (RF), representing re-sensitizing activity, was defined as the IC_50_ value for the paclitaxel-only group divided by the IC_50_ value of the co-treatment. The results are shown in Table 3. Reversal folds among all derivatives are organized in Figure 3. It can be seen that the most potent compounds are **8**, **12** and **26** with RF values of 666, 692, and 348, respectively.

In general, methyl ester derivatives have better collateral activity, manifested in the form of the lower doses (5 μM and 10 μM) used in the methyl ester group, while the higher doses (10 μM and 20 μM) were used in the acid group. In HeLa S3 cell group, none of the derivatives exhibited a collateral sensitivity effect, as reflected in the low RF values. On the other hand, the co-treatment group significantly improved the anti-proliferative effect of paclitaxel in KBvin cells. In order to obtain more structural insights into collateral sensitivity, the detailed structure–activity relationship is discussed in terms of the anti-proliferative effects of the triterpene functional groups in KBvin cells at 10 μM.

In the R_1_ position, the carbonyl group contributed extensively to the collateral sensitivity effect. The two most potent compounds were **8** and **12**, with IC_50_ co-treatment values of 1.75 nM and 1.69 nM, respectively, which convert to RF values of 666 and 692. Additionally, for all compounds with α-hydroxy in the R_1_ position, **16**, **26** and **28** had the strongest inhibitory effects, with RF values of 236, 348 and 297, respectively. Substitution of α-*O*-formate in the R_1_ position reduced chemoreversal ability, for example, compound **18** with an RF value of 44. In the R_2_ position, β-methyl decreased the chemoreversal effects, which was verified by the low RF values in **15** (RF of 1.62). However, compound **6**, with an α-methyl substituent, exhibited better chemoreversal activity than was the case for **15**. (RF values of 64.02 and 1.62, respectively). Therefore, we suspect that the configuration at the R_2_ position plays an important role in target protein binding. In the R_3_ position, the α-hydroxyl and β-methyl groups reduced inhibitory activity, for example, **14** and **22** with RF values of ~4 and 10, respectively. Interestingly, compound **22** with a β-methyl substitution had better chemoreversal ability at low co-treatment concentrations, for which the RF value was 66 at 5 μM and 11 μM at 10 μM. Moreover, β-hydroxyl retained its chemoreversal ability, for example, compound **26** with an RF of 349. These results indicate that the target protein binding site may be related to the hydrogen bonding with β-OH at the R_3_ position. In addition, of the compounds **23**, **24**, **27** and **28** with the hydrogen group at the R_3_ position, **28** was the most effective (RF of 297). The carbonyl substituted at R_3_ retained its inhibitory activity among most of the derivatives, such as compounds **8** and **16** (RF values of 666 and 236, respectively). In the R_4_ position, only hydrogen substitution led to better chemoreversal ability than that when using the α-hydroxyl substitution. For example, **12** manifested stronger inhibitory activity than **10** (RF values of 692 and 22, respectively). In the R_5_ position, as previously predicted, the ester derivatives exhibited better chemoreversal ability than their acid derivatives, evidenced by compound pairs **7** and **8** (RF values of ~5 and 666, respectively) and compound pairs **11** and **12** (RF values of 4 and 692, respectively).

**Figure 4:**
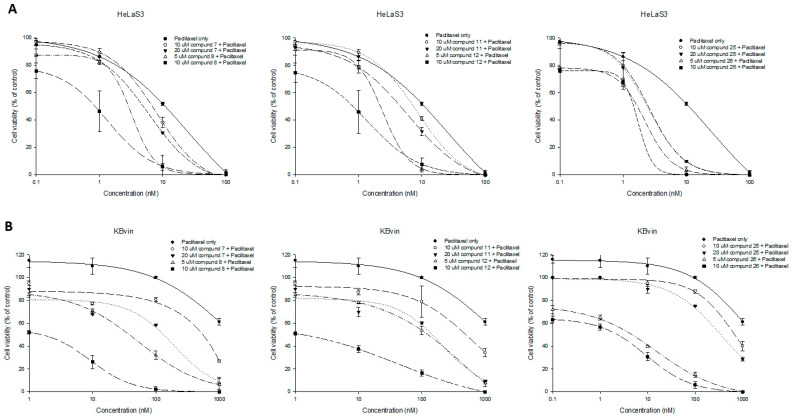
Dose–response curves of compounds **7**–**8**, **11**–**12**, and **25**–**26** in HeLa S3 (**A**) and KBvin (**B**).

The dose–response curves of the most active compound pairs **7**–**8**, **11**–**12**, and **25**–**26** are shown in Figure 4. Cell viability was measured along with different concentrations of paclitaxel combined with triterpenes. Paclitaxel resistance in KBvin cells can be observed from 70–80% cell survival at a very high concentration of paclitaxel (1000 nM), where HeLa was all died at 100 nM. The higher dose of the test compounds inhibited more cell growth in HeLa S3 and KBvin. The ester derivatives (**8**, **12**, **26**) were more potent than its acid (**7**, **11**, **25**), especially in MDR KBvin cells. Moreover, ester derivatives (**8**, **12**, **26**) exhibited significant collateral sensitivity, evidenced by the extensive curve shift from the paclitaxel-only group, especially in MDR KBvin cells. The most potent compounds **8** and **12** at 10 μM combined with paclitaxel at 1 nM can even kill 50% MDR KBvin cells, significantly re-sensitizing the effect of paclitaxel.

**Figure 5:**
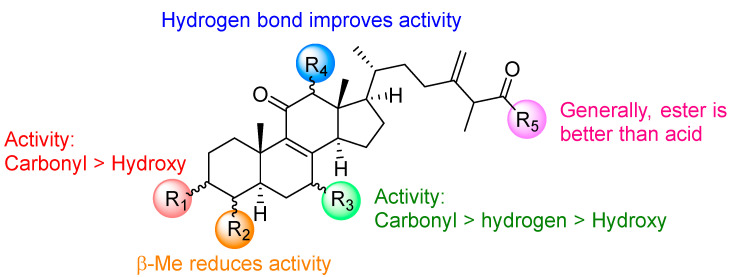
Summary of the SAR of all triterpenes in terms of collateral sensitivity.

A summary of the structure–activity relationship (SAR) of triterpene derivatives are shown in Figure 5. In the R_1_ and R_3_ positions, the carbonyl substituent exhibited better chemoreversal ability than the other substituents. When the α-methyl substituent is epimerized to β-methyl in the R_2_ position, the inhibitory activity in KBvin cells was reduced. In the R_4_ position, the hydrogen bond improved the collateral sensitivity. Finally, the R_5_ ester derivatives were found to be more potent than their acid derivatives.

### 2.4. Research on the Mechanism of the Most Active Compounds

**Figure 6:**
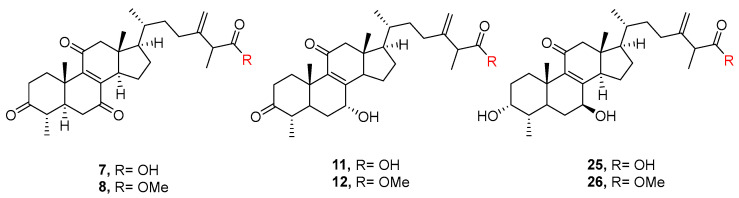
Structures of compounds **7**–**8**, **11**–**12**, and **25**–**26**.

**Figure 7:**
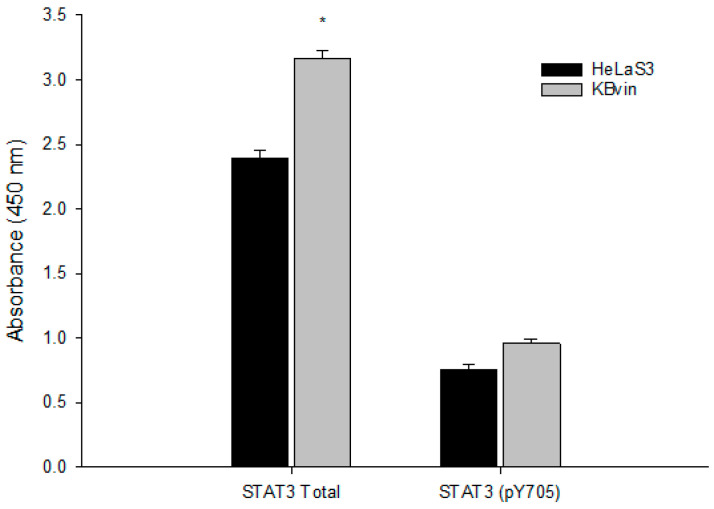
Baseline expression of STAT3 total and STAT3 phosphorylation in HeLaS3 and KBvin. * denotes *p* < 0.05 as compared to the vehicle control.

**Figure 8:**
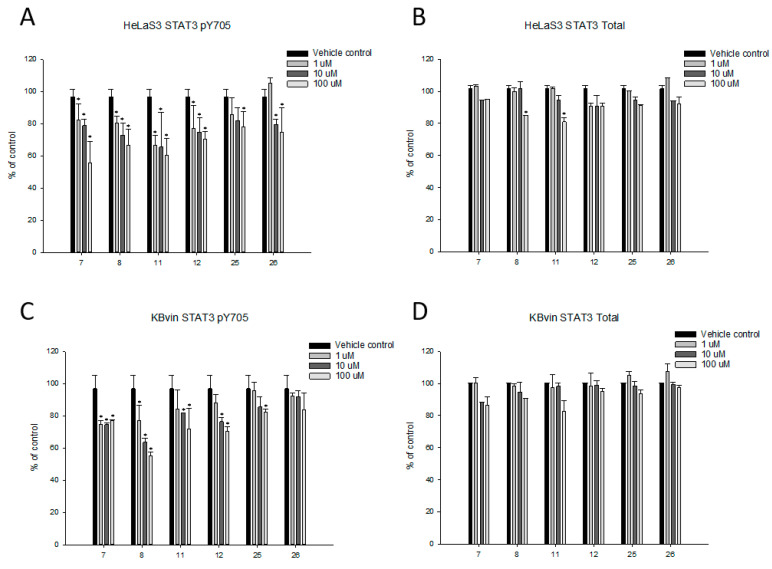
Effects of the testing compounds on the expression of STAT3 phosphorylated at Tyr705 and total STAT3 proteins in the HeLaS3 cells (**A**,**B**) and KBvin cells (**C**,**D**). * denotes *p* < 0.05 as compared to the vehicle control.

The multidrug resistance mechanisms of the most potent compounds (**8**, **12** and **26**) were studied, and their acid derivatives (**7**, **11** and **25**) were also compared (Figure 6). Expression of total STAT3 was significantly higher in resistant KBvin cells than in drug-sensitive HeLaS3 cells, which means STAT3 phosphorylation was one of the resistance mechanism pathways. (Figure 7) Inhibition of SATA3 could be a strategy for chemoreversal activity in KBvin. According to previous research results indicating that zhankuic acid A can block the phosphorylation of STAT3 [13], we decided to treat the derivatives of both HeLa and KBvin cells in order to study the expression of phosphorylated STAT3 (Figure 8).

The expression of phosphorylated STAT3 was significantly inhibited by the testing compounds in both drug-sensitive HeLaS3 and drug-resistant KBvin cells, whereas the expression of total STAT3 was not significantly influenced in KBvin cells. These results indicated that the testing compounds influenced the activation of STAT3, which in turn contributed to the collateral sensitivity of the triterpenes.

## 3. Materials and Methods

### 3.1. General

All chemicals were obtained from Merck or Sigma-Aldrich. The chemical reaction was monitored using thin-layer chromatography (TLC) using silica gel 60 F254-pre-coated glass plates with a thickness of 0.25 mm and a UV lamp to visualize the plate. Column chromatography was performed using silica gel (230–400 mesh). Optical rotations of the final compounds were measured with a Jasco P-2000 digital polarimeter. The infrared (IR) spectra were obtained with a Jasco FT/IR-4000 FTIR spectrometer. The NMR spectra were recorded on commercial instruments (Bruker AV 500 FT-NMR spectrometer). Low-resolution and high-resolution mass spectra were performed using Fourier-transfer mass spectrometry (FT-MS). Mass spectra were recorded in both positive modes with Bruker APEX II (National Sun Yat-sen University). Acid natural products were isolated from Prof. Tian-Shung Wu’s lab (Tainan, NCKU, Taiwan) [25,27,32,33].

### 3.2. General Procedure for Methylation

The starting material (0.01 g, 0.02 mmol), potassium carbonate (0.05 g, 0.04 mmol), iodomethane (0.04 g, 0.03 mmol), and acetone (1 mL) were all mixed in a round-bottomed flask. Then, the mixture was heated to reflux, and the reaction was monitored using thin-layer chromatography. After completion of the reaction, the mixture was removed with acetone by vacuuming, followed by extraction with water and ethyl acetate. The organic layer was collected, dried over anhydrous MgSO_4_, and concentrated under vacuum conditions. The reaction mixture was purified through silica gel column chromatography using solvent system ethyl acetate/ hexane to obtain the methylation product. The ^1^H and ^13^C NMR spectra of compounds were provided in Figures S1–S24.

### 3.3. Methyl 3α,7α,12α-Trihydroxy-4α-methylergosta-8,24(28)-dien-11-on-26-oate (**6**)

Colorless solid. Yield: 92%. [α]*_D_*^25^ + 86 (*c* 0.0006, MeOH). UV (MeOH) λ _max_ (log *ε*) 255 nm. IR (KBr) ν_max_ 3464, 2954, 1652, 715 cm^−1^. Mp: 207 °C. ^1^H NMR (500 MHz, CDCl_3_, ppm) δ 4.91 (d, *J* = 7.8 Hz, 1H, CH_2_-a), 4.87 (d, *J* = 7.2 Hz, 1H, CH_2_-b), 4.07 (d, *J* = 3.8 Hz, 1H, CH), 3.96 (s, 1H, CH), 3.82 (d, *J* = 2.4 Hz, 1H, CH), 3.67 (d, *J* = 1.2 Hz, 3H, OCH_3_), 3.64 (br, 1H, OH), 3.12 (m, 2H, CH_2_), 2.34 (m, 1H, CH), 2.12, (m, 2H, CH_2_), 1.96 (m, 4H, CH_2_), 1.78 (m, 3H, CH, CH_2_), 1.69 (m, 10H, CH_3_, CH_2_, CH), 1.45 (m, 3H, CH_2_, CH), 1.35, (m, 1H, CH), 1.28 (dd, *J* = 7.5, 3.2 Hz, 3H, CH_3_), 1.05 (s, 3H, CH_3_), 0.96 (t, *J* = 6.8 Hz, 6H, CH_3_). ^13^C NMR (125 MHz, CDCl_3_, ppm) δ 200.5, 175.1, 153.6, 148.7, 148.6, 139.2, 110.9, 81.3, 71.7, 66.1, 51.9, 48.5, 46.7, 45.8, 45.5, 44.0, 37.1, 35.8, 35.6, 33.8, 31.5, 29.2, 28.3, 26.7, 22.4, 17.8, 16.5, 16.4, 16.2, 11.3. HR-ESI-MS *m*/*z* 525.3185 (M+Na)^+^ (calcd. for C_30_H_46_O_6_Na, 525.3185).

### 3.4. Zhankuic Acid A Methyl Ester (**8**)

Colorless solid. Yield: 91%. [α]*_D_*^25^ + 23 (*c* 0.0006, MeOH). UV (MeOH) λ _max_ (log *ε*) 265 nm. IR (KBr) ν_max_ 2935, 2874, 1676, 1162 cm^−1^. Mp: 112 °C. ^1^H NMR (500 MHz, CDCl_3_, ppm) δ 4.92 (d, *J* = 6.7 Hz, 1H, CH_2_-a), 4.87 (d, *J* = 6.7 Hz, 1H, CH_2_-b), 3.67 (d, *J* = 1.4 Hz, 3H, OCH_3_), 3.13 (q, *J* = 13.7, 6.9 Hz, 1H, CH), 3.07 (m, 1H, CH), 2.93 (d, *J* = 14.0 Hz, 1H, CH), 2.66 (m, 1H, CH), 2.54 (m, 3H, CH_2_, CH), 2.43 (m, 4H, CH_2_, CH), 2.11 (m, 1H, CH), 1.92 (m, 2H, CH_2_), 1.53 (s, 3H, CH_3_), 1.42 (m, 5H, CH_2_, CH), 1.28 (dd, *J* =7.1, 2.9 Hz, 3H, CH_3_), 1.05 (d, *J* = 6.6 Hz, 3H, CH_3_), 0.94 (d, *J* = 5.7 Hz, 3H, CH_3_), 0.70 (s, 3H, CH_3_). ^13^C NMR (125 MHz, CDCl_3_, ppm) δ 210.6, 202.6, 200.8, 175.0, 152.0, 145.5, 111.0, 57.3, 54.0, 51.9, 49.3, 48.9, 47.1, 45.6, 44.0, 39.0, 38.3, 37.6, 35.6, 34.7, 33.8, 31.3, 31.0, 27.8, 24.9, 18.5, 16.5, 16.3, 12.00, 11.4. HR-ESI-MS *m*/*z* 505.2925 (M+Na)^+^ (calcd. for C_30_H_42_O_5_Na, 505.2925).

### 3.5. Methyl 7α, 12α-Dihydroxy-3,11-dioxo-4α-methylergosta-8,24(28)-dien-26-oate (**10**)

Colorless solid. Yield: 91%. [α]*_D_*^25^ + 155 (*c* 0.0006, MeOH). UV (MeOH) λ _max_ (log *ε*) 253 nm. IR (KBr) ν_max_ 3452, 1647, 1367, 702 cm^–1^. Mp: 238 °C. ^1^H NMR (500 MHz, CDCl_3_, ppm) δ 4.93 (d, *J* = 7.4 Hz, 1H, CH_2_-a), 4.92 (d, *J* = 7.4 Hz, 1H, CH_2_-b), 4.18 (br, 1H, OH), 4.02 (s, 1H, CH), 3.70 (s, 3H, OCH_3_), 3.12 (m, 1H, CH), 3.09 (m, 1H, CH), 2.89 (m, 1H, CH), 2.65 (br, 1H, OH), 2.53 (m, 1H, CH), 2.40 (m, 2H, CH), 2.05 (m, 4H, CH_2_, CH), 1.66 (m, 7H, CH_2_, CH), 1.53 (m, 1H, CH), 1.42 (m, 3H, CH_2_, CH), 1.31 (m, 6H, CH_2_, CH), 1.26 (m, 1H, CH), 1.20 (m, 1H, CH), 1.09 (d, *J* = 6.8 Hz, 3H, CH_3_), 0.97 (d, *J* = 6.8 Hz, 3H, CH_3_), 0.70 (s, 3H, CH_3_). ^13^C NMR (125 MHz, CDCl_3_, ppm) δ 212.8, 199.8, 175.1, 153.5, 148.4, 138.1, 111.0, 81.0, 65.9, 51.9, 48.4, 46.9, 45.6, 44.4, 43.9, 43.7, 37.6, 36.8, 35.5, 34.4, 33.7, 31.4, 30.6, 26.5, 22.3, 17.78, 16.5, 16.3, 11.7, 11.3. HR-ESI-MS *m*/*z* 523.3029 (M+Na)^+^ (calcd. for C_30_H_44_O_6_Na, 523.3029).

### 3.6. Methyl-7β-hydroxy-3,11-dioxo-4α-methylergosta-8,24(28)-dien-26-oate (**12**)

Pale yellow solid. Yield: 87%. [α]*_D_*^25^ + 139 (*c* 0.0006, MeOH). UV (MeOH) λ _max_ (log *ε*) 250 nm. IR (KBr) ν_max_ 3455, 2998, 2750, 1560 cm^−1^. Mp: 197 °C. ^1^H NMR (500 MHz, CDCl_3_, ppm) δ 4.94 (d, *J* = 6.6 Hz, 1H, CH_2_-a), 4.90 (d, *J* = 5.5 Hz, 1H, CH_2_-b), 4.28 (br, 1H, OH), 3.70 (s, 3H, OCH_3_), 3.16 (q, *J* = 13.3, 7.20 Hz, 1H, CH), 3.08 (m, 1H, CH), 2.85 (m, 2H, CH), 2.51 (m, 3H, CH_2_, CH), 2.39 (m, 4H, CH_2_, CH), 2.07 (m, 4H, CH_2_, CH), 1.88, (d, *J* = 13.3 Hz, 2H, CH_2_), 1.69 (m, 5H, CH_2_, CH), 1.31 (m, 6H, CH_3_), 1.09(m, 4H, CH_3_, CH), 0.95 (d, *J* = 5.9 Hz, 3H, CH_3_), 0.73 (s, 3H, CH_3_). ^13^C NMR (125 MHz, CDCl_3_, ppm) δ 212.9, 200.9, 175.0, 153.0, 148.5, 140.7, 111.0, 66.1, 57.6, 55.1, 51.9, 51.2, 47.1, 45.6, 44.6, 43.8, 37.6, 37.2, 35.8, 34.6, 33.8, 31.3, 30.7, 27.5, 23.1, 18.4, 16.5, 16.3, 11.9, 11.8. LR-ESI-MS *m*/*z* (rel. int.) 507 (M+Na)^+^.

### 3.7. Methyl 3α,4β,7β-Trihydroxyergosta-8,24(28)-dien-11-on-26-oate (methyl antcamphorol D, **14**)

Colorless solid. Yield: 88%. [α]*_D_*^25^ + 90 (*c* 0.0006, MeOH). UV (MeOH) λ _max_ (log *ε*) 255 nm. IR (KBr) ν_max_ 3340, 1635, 718 cm^–1^. Mp: 273 °C. ^1^H NMR (500 MHz, Acetone-d_6_, ppm) δ 4.89 (m, 2H, CH_2_), 4.37 (q, *J* = 6.7 Hz, 1H, CH), 3.66 (s, 3H, OCH_3_), 3.6 (m, 1H, CH), 3.44 (m, 1H, CH), 3.20 (q, *J* = 6.76 Hz, 1H, CH), 2.77 (m, 1H, CH), 2.68 (d, *J* = 13.7 Hz, 1H, CH), 2.44 (m, 2H, CH), 2.19 (m, 4H, CH_2_, CH), 2.08 (m, 1H, CH), 1.97 (m, 3H, CH_2_, CH), 1.81 (m, 1H, CH), 1.65 (m, 1H, CH), 1.54 (m, 1H, CH), 1.48 (m, 1H, CH), 1.45 (m, 4H, CH_3_, CH), 1.35 (m, 3H, CH_2_, CH), 1.25 (t, *J* = 3.9 Hz, 2H, CH_2_), 1.24 (s, 1H, CH), 1.23 (s, 3H, CH_3_), 0.96 (m, 3H, CH_3_), 0.76 (s, 3H, CH_3_). ^13^C NMR (125 MHz, CDCl_3_, ppm) δ 210.8, 202.8, 178.0, 177.5, 175.0, 148.6, 143.9, 110.9, 74.3, 73.8, 69.5, 57.6, 53.8, 51.9, 49.6, 47.4, 45.8, 43.3, 39.3, 35.6, 31.7, 29.3, 27.5, 27.0, 25.4, 25.1, 18.6, 16.5, 12.0. HR-ESI-MS *m*/*z* 525.3187 (M+Na)^+^ (calcd. for C_30_H_46_O_6_Na, 525.3187).

### 3.8. Methyl 3α,12α-Dihydroxy 4α-methylergosta-8,24(28)-diene-7,11-dion-26-oate (methyl antcinate H, **16**)

Pale yellow solid. Yield: 85%. [α]*_D_*^25^ + 102 (*c* 0.0006, MeOH). UV (MeOH) λ _max_ (log *ε*) 275 nm. IR (KBr) ν_max_ 3592, 2908, 2336, 1670 cm^–1^. Mp: 170 °C. ^1^H NMR (500 MHz, CDCl_3_, ppm) δ 4.91 (d, *J* = 7.7 Hz, 1H, CH_2_-a), 4.87 (d, *J* = 6.6 Hz, 1H, CH_2_-b), 4.05 (s, 1H, CH), 3.79 (d, J = 2.4 Hz, 1H, CH), 3.67, (d, *J* = 1.9 Hz, 3H, OCH_3_), 3.13 (dd, *J* = 14.6, 7.1 Hz, 1H, CH), 3.00 (dd, *J* = 13.0, 7.3 Hz, 1H, CH), 2.75 (br, 1H, OH), 2.53 (m, 1H, CH), 2.42 (dd, *J* = 15.5, 2.7 Hz, 1H, CH), 2.36 (m, 1H, CH), 2.23 (t, *J* = 15.2 Hz, 1H, CH), 2.13 (m, 2H, CH_2_), 1.90 (m, 5H, CH_3,_ CH), 1.73 (m, 2H, CH_2_), 1.44 (m, 4H, CH_3_, CH), 1.29 (s, 3H, CH_3_), 1.28 (d, *J* = 3.0 Hz, 2H, CH_2_), 1.27 (d, *J* = 3.0 Hz, 2H, CH_2_), 0.96 (t, *J* = 6.9 Hz, 6H, CH_3_), 0.64 (s, 3H, CH_3_). ^13^C NMR (125 MHz, CDCl_3_, ppm) δ 202.4, 201.6, 175.1, 152.2, 148.5, 144.6, 110.9, 80.8, 70.4, 51.9, 49.5, 45.6, 41.8, 40.7, 38.3, 38.1, 35.4, 34.5, 33.9, 31.3, 31.1, 27.9, 27.8, 26.9, 23.9, 17.9, 16.3, 16.1, 15.6, 11.5. LR-ESI-MS *m*/*z* (rel. int.) 523 (M+Na)^+^.

### 3.9. Zhankuic Acid Methyl Ester C 3-O-formate (**18**)

Yellow solid. Yield: 75%. [α]*_D_*^25^ + 65 (*c* 0.0006, MeOH). UV (MeOH) λ _max_ (log *ε*) 271 nm. IR (KBr) ν_max_ 2355, 1683, 1546 cm^–1^. Mp: 169 °C. ^1^H NMR (500 MHz, CDCl_3_, ppm) δ 8.08 (s, 1H, CHO), 5.10 (d, *J* = 2.4 Hz, 1H, CH), 4.92 (d, *J* = 7.2 Hz, 1H, CH_2_-a), 4.87 (d, *J* = 6.7 Hz, 1H, CH_2_-b), 4.09 (d, *J* = 3.9 Hz, 1H, CH), 3.67 (d, *J* = 1.7 Hz, 3H, OCH_3_), 3.13 (dd, *J* = 13.9, 7.2 Hz, 1H, CH), 3.02 (dd, *J* = 12.0, 7.7 Hz, 1H, CH), 2.54 (m, 1H, CH), 2.47 (m, 2H, CH_2_), 2.25 (m, 2H, CH_2_), 2.13 (m, 2H, CH_2_), 1.92 (m, 6H, CH_2_, CH), 1.46 (m, 2H, CH_2_), 1.37 (m, 1H, CH), 1.33 (s, 3H, CH_3_), 1.28 (dd, *J* = 7.1, 3.0 Hz, 3H, CH_3_), 1.24 (m, 1H, CH), 0.97 (d, *J* = 6.5 Hz, 3H, CH_3_), 0.89 (d, *J* = 7.0 Hz, 3H, CH_3_), 0.65 (s, 3H, CH_3_). ^13^C NMR (125 MHz, CDCl_3_, ppm) δ 201.8, 201.1, 175.0, 160.7, 151.7, 148.5, 144.8, 111.0, 80.7, 73.1, 51.9, 49.5, 45.7, 45.6, 41.9, 38.0, 35.4, 33.8, 33.2, 31.3, 31.1, 28.4, 26.9, 26.3, 23.9, 17.9, 16.5, 16.3, 16.1, 15.2, 11.5. HR-ESI-MS *m*/*z* 551.2976 (M+Na)+ (calcd. for C31H44O7Na, 551.2976).

### 3.10. Methyl 3α-Hydroxy-7,11-dioxo-4α-methylergosta-8,24(28)-dien-26-oate (**20**)

Yellow solid. Yield: 82%. [α]*_D_*^25^ + 166 (*c* 0.0006, MeOH). UV (MeOH) λ _max_ (log *ε*) 265 nm. IR (KBr) ν_max_ 3439, 2955, 2362, 1667 cm^−1^. Mp: 107 °C. ^1^H NMR (500 MHz, CDCl_3_, ppm) δ 4.94 (d, *J* = 6.8 Hz, 1H, CH_2_-a), 4.89 (d, *J* = 6.2 Hz, 1H, CH_2_-b), 3.81 (br, 1H, OH), 3.69 (d, *J* = 2.0 Hz, 3H, OCH_3_), 3.16 (dd, *J* = 14.1, 7.2 Hz, 1H, CH), 2.91 (d, *J* = 13.5 Hz, 1H, CH), 2.65 (dd, *J* = 12.4, 7.5 Hz, 1H, CH), 2.54 (m, 2H, CH_2_), 2.43 (m, 2H, CH), 2.27 (t, *J* = 15.3 Hz, 1H, CH), 2.14 (m, 2H, CH), 1.93 (m, 4H, CH_2_, CH), 1.75 (m, 2H, CH_2_), 1.44 (m, 4H, CH_3_, CH), 1.33 (s, 3H, CH_3_), 1.30 (d, *J* = 2.8 Hz, 2H, CH_2_), 1.29 (d, *J* = 2.8 Hz, 2H, CH_2_), 1.27 (m, 1H, CH), 0.98 (d, *J* = 7.1 Hz, 2H, CH_2_), 0.95 (d, *J* = 5.7 Hz, 2H, CH_2_), 0.69 (s, 3H, CH_3_). ^13^C NMR (125 MHz, CDCl_3_, ppm) δ 202.9, 175.0, 153.8, 148.6, 144.7, 110.9, 110.9, 70.3, 57.5, 53.9, 51.9, 49.5, 47.3, 45.8, 45.6, 41.1, 38.7, 38.1, 35.6, 34.5, 33.8, 31.2, 31.3, 29.1, 27.9, 25.0, 18.5, 16.3, 15.9, 12.0. LR-ESI-MS *m*/*z* (rel. int.) 507 (M+Na)^+^.

### 3.11. Methyl 7β-Hydroxy-3,11-dioxo-4α-methylergosta-8,24(28)-dien-26-oate (**22**)

White solid. Yield: 79%. [α]*_D_*^25^ + 164 (*c* 0.0006, MeOH). UV (MeOH) λ _max_ (log *ε*) 252 nm. IR (KBr) ν_max_ 3354, 2872, 1720, 1636 cm^–1^. Mp: 188 °C. ^1^H NMR (500 MHz, CDCl_3_, ppm) δ 4.93 (d, *J* = 6.9 Hz, 1H, CH_2_-a), 4.90 (d, *J* = 6.9 Hz, 1H, CH_2_-b), 4.42 (t, *J* = 8.1 Hz, 1H, CH), 3.70 (s, 3H, OCH_3_), 3.15 (dd, *J* = 14.3, 7.1 Hz, 1H, CH), 3.00 (m, 1H, CH), 2.86 (d, *J* = 14.0 Hz, 1H, CH), 2.73 (dd, *J* = 12.3, 6.0 Hz, 1H, CH), 2.52 (m, 1H, CH), 2.37 (m, 3H, CH_2_, CH), 2.29 (m, 1H, CH), 2.11 (m, 1H, CH), 1.96 (m, 3H, CH_2_, CH), 1.56 (m, 5H, CH_2_, CH), 1.47 (s, 3H, CH_3_), 1.41 (m, 4H, CH_2_, CH), 1.30 (dd, *J* = 7.1, 3.17 Hz, 3H, CH_3_), 1.06 (d, *J* = 6.9 Hz, 3H, CH_3_), 0.95 (d, *J* = 5.9 Hz, 3H, CH_3_), 0.81 (s, 3H, CH_3_). ^13^C NMR (125 MHz, CDCl_3_, ppm) δ 202.9, 175.0, 153.8, 148.6, 144.7, 110.9, 110.9, 70.3, 57.5, 53.9, 51.9, 49.5, 47.3, 45.8, 45.6, 41.1, 38.7, 38.1, 35.6, 34.5, 33.9, 31.2, 31.0, 29.1, 27.9, 25.0, 18.5, 16.3, 15.9, 12.0. LR-ESI-MS *m*/*z* (rel. int.) 507 (M+Na)^+^.

### 3.12. Methyl 4α-Methylergosta-8,24(28)-diene-3,11-dion-26-oate (**24**)

Yellow powder. Yield: 82%. [α]*_D_*^25^ + 164 (*c* 0.0006, MeOH). UV (MeOH) λ _max_ (log *ε*) 251 nm. IR (KBr) ν_max_ 3608, 2957, 2356, 1706 cm^–1^. Mp: 105 °C. ^1^H NMR (500 MHz, CDCl_3_, ppm) δ 4.93 (d, *J* = 7.2 Hz, 1H, CH_2_-a), 4.90 (d, *J* = 6.8 Hz, 1H, CH_2_-b), 3.70 (s, 3H, OCH_3_), 3.17 (m, 2H, CH), 2.81 (d, *J* = 14.7 Hz, 1H, CH), 2.64 (m, 1H, CH), 2.53 (m, 1H, CH), 2.37 (m, 4H, CH_2_, CH), 2.17 (m, 3H, CH), 1.94 (m, 3H, CH_2_, CH), 1.81 (m, 2H, CH_2_), 1.51 (m, 6H, CH_2_, CH), 1.40 (m, 3H, CH_2_, CH), 1.35 (m, 4H, CH_3_, CH), 1.30 (dd, *J* = 3.1, 1.8 Hz, 3H, CH_3_), 1.27 (m, 1H, CH), 1.06 (d, *J* = 6.4 Hz, 3H, CH_3_), 0.94 (d, *J* = 6.0 Hz, 3H, CH_3_), 0.74 (s, 3H, CH_3_). ^13^C NMR (125 MHz, CDCl_3_, ppm) δ 213.3, 199.9, 175.0, 157.0, 148.7, 148.5, 138.7, 110.9, 57.8, 55.2, 53.0, 51.9, 50.6, 47.2, 45.6, 44.3, 37.8, 36.6, 35.7, 35.1, 33.8, 31.3, 30.2, 27.5, 23.7, 20.9, 18.3, 17.4, 16.3, 11.3. LR-ESI-MS *m*/*z* (rel. int.) 491 (M+Na)^+^.

### 3.13. Methyl 3α,7β-Dihydroxy-4α-methylergosta-8,24(28)-dien-11-on-26-oate (**26**)

Yellow solid. Yield: 84%. [α]*_D_*^25^ + 156 (*c* 0.0006, MeOH). UV (MeOH) λ _max_ (log *ε*) 254 nm. IR (KBr) ν_max_ 3399, 2941, 1721, 1648 cm^−1^. Mp: 197 °C. ^1^H NMR (500 MHz, CDCl_3_, ppm) δ 4.90 (d, *J* = 7.4 Hz, 1H, CH_2_-a), 4.87 (d, *J* = 6.7 Hz, 1H, CH_2_-b), 4.36 (m, 1H, CH), 3.75 (d, J = 2.5 Hz, 1H, CH), 3.67 (d, *J* = 0.8 Hz, 3H, OCH_3_), 3.13 (dd, *J* = 14.2, 7.1 Hz, 1H, CH), 2.80 (d, *J* = 13.6 Hz, 1H, CH), 2.67 (m, 1H, CH), 2.42 (m, 1H, CH), 2.34 (d, *J* = 13.6 Hz, 1H, CH), 2.02 (m, 6H, CH_2_, CH), 1.79 (m, 1H, CH), 1.66 (m, 1H, CH),1.58 (m, 2H, CH_2_), 1.53 (m, 2H, CH_2_), 1.60 (m, 2H, CH_2_), 1.53 (m, 1H, CH), 1.30 (m, 1H, CH), 1.27 (m, 4H, CH_3_, CH), 1.21 (s, 3H, CH_3_), 0.98 (d, *J* = 6.8 Hz, 3H, CH_3_), 0.94 (d, *J* = 5.7 Hz, 3H, CH_3_), 0.77 (s, 3H, CH_3_). ^13^C NMR (125 MHz, CDCl_3_, ppm) δ 201.6, 175.0, 152.2, 148.7, 143.3, 110.8, 70.9, 70.4, 58.2, 54.4, 53.3, 51.9, 47.7, 45.8, 39.7, 37.2, 35.8, 34.2, 34.0, 31.5, 31.1, 29.3, 28.7, 27.9, 24.9, 18.5, 17.4, 16.5, 16.2, 12.1. HR-ESI-MS *m*/*z* 487.6417 (M+H)+ (calcd. for C_30_H_47_O_5_, 487.3417).

### 3.14. Methyl 3β,12β-Dihydroxy-11-oxo-4β-methylergosta-8,24(28)-dien-26-oate (methyl antcin M, **28**)

Yellow solid. Yield: 87%. [α]*_D_*^25^ + 70 (*c* 0.0006, MeOH). UV (MeOH) λ _max_ (log *ε*) 258 nm. IR (KBr) ν_max_ 2942, 2359, 1728, 1652 cm^−1^. Mp: 170 °C. ^1^H NMR (500 MHz, CDCl_3_, ppm) δ 4.91 (d, *J* = 7.2 Hz, 1H, CH_2_-a), 4.87 (d, *J* = 6.8 Hz, 1H, CH_2_-b),4.09 (m, 1H, CH), 3.82 (m, 1H. CH) 3.70 (d, *J* = 1.6 Hz, 3H, OCH_3_), 3.16 (m, 1H, CH), 3.03 (m, 1H, CH), 2.55 (m, 1H, CH), 2.43 (m, 1H, CH), 2.30 (m, 1H, CH), 2.20 (m, 2H, CH_2_), 2.17 (m, 2H, CH_2_), 1.93 (m, 4H, CH_2_, CH), 1.85 (m, 2H, CH_2_), 1.76 (m, 4H, CH_2_, CH), 1.48 (m, 5H, CH_2_, CH), 1.33 (s, 3H, CH_3_), 1.30 (dd, *J* =7.1, 2.9 Hz, 3H, CH_3_), 0.99 (t, *J* = 6.5 Hz, 6H, CH_3_), 0.67 (m, 3H, CH_3_). ^13^C NMR (125 MHz, CDCl_3_, ppm) δ 201.6, 175.1, 152.2, 148.5, 144.6, 110.9, 80.7, 70.3, 51.9, 49.5, 45.6, 45.5, 41.8, 40.7, 38.3, 38.1, 35.4, 34.5, 33.9, 31.3, 31.1, 28.9, 27.8, 26.9, 23.9, 17.9, 16.3, 16.0, 15.6, 11.5. LR-ESI-MS *m*/*z* (rel. int.) 509 (M+Na)^+^.

### 3.15. Culture of Cell Lines

Bioresource Collection and Research Center (Hsinchu, Taiwan) supplied the human cervical epithelioid carcinoma cell line HeLaS3. Dr. Kuo-Hsiung Lee (University of North Carolina, Chapel Hill, NC, USA) kindly gave multi-drug resistant human cervical cancer cell line KBvin to us. All cancer cell lines were cultured in RPMI-1640 containing 10% FBS at 37 °C in a humidified atmosphere of 5% CO_2_.

### 3.16. SRB Cytotoxicity Assay and Reversal Fold Calculation

The cells were treated with a series of concertation of chemotherapeutic agents and combined without or with test compounds after 72 h; then 50% trichloroacetic acid (TCA) was charged to fix the cell for 30 min. After air-drying, followed by 0.04% SRB stained for 30 min, and 1% acetic acid washing, 10 mM Tris base was applied to dissolve the bound stain, and the absorbance was measured using the BioTek Synergy HT Multi-Mode Microplate Reader at 515 nm. Reversal folds were calculated by dividing the IC_50_ of chemotherapeutic drug only by the IC_50_ of compound–chemotherapeutic drug combination treatment.

### 3.17. Enzyme-Linked Immunosorbent Assay (ELISA)

Semiquantitative measurements of STAT3 phosphorylated at Tyr705 and total STAT3 proteins in cell lysates were performed using the STAT3 (pY705) + Total ELISA Kit (Abcam, Cambridge, CB2 0AX, UK) according to the manufacturer’s instructions. Briefly, 1 × 10^5^ cells/well were seeded in a 6-well plate and treated with the test compounds for 24 h. The cells were solubilized using a chilled 1× cell extraction buffer PTR, and the sample protein concentrations were determined using a BCA protein assay (Thermo Fisher, Waltham, MA, USA). Samples were diluted to 300 ng/μL in a 1x cell extraction buffer PTR, and 50 μL of all samples and controls were added to the appropriate wells. Then, 50 μL of the antibody cocktail was added to each well. The plate was sealed and shaken at 400 rpm for 1 h at room temperature. The plate was further washed three times with a wash buffer. After removing any excess liquid, 100 μL of a TMB substrate were added to each well, after which the samples were incubated for 15 min in the dark while being shaken at 400 rpm. The plate was read at an OD of 450 nm after adding 100 μL of a stop solution to each well.

## 4. Conclusions

A total of 12 triterpene derivatives were synthesized, and chemoreversal ability tests were conducted. Among all of the derivatives, the RF values of 8 and 12 could be as much as 600 times higher than that for the paclitaxel group in KBvin cells, whereas the IC50 values for 8 and 12 were similar to those of the paclitaxel-only treatment group in HeLa S3 cells, indicating a strong collateral sensitivity effect. On the other hand, the resistance mechanism study of compounds 8, 12, and 26 showed that the derivatives can inhibit the ability of phosphorylation of STAT3 to poison resistant cancer cells. These results may provide a new combined therapy with paclitaxel to treat multidrug-resistant cancers and provide new therapy options for cancer patients.

## Figures and Tables

**Table 1:** Name and reference of zhankuic acid-type compounds.

No	Name	Reference
**5**	Salmonone D	[27]
**7**	Antcin B	[32]
**9**	Salmonone E	[27]
**11**	7α-hydroxy-3,11-dioxo-4α-methylergosta-8,24(28)-dien-26-oic acid	[25]
**13**	Antcin K	[33]
**15**	Zhankuic acid C	[25]
**17**	Zhankuic acid C 3-*O*-formate	[27]
**19**	Zhankuic acid B	[32]
**21**	Antcin C	[32]
**23**	Antcin A	[25]
**25**	Camphoratin B	[25]
**27**	3α,12α-dihydroxy-4α-methylergosta-8,24(28)-dien-11-on-26-oic acid	[33]

**Table 2:**
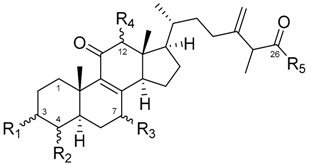
Cytotoxicity IC_50_ of **5**–**28** compounds.

No	Structure					KBvin	HeLa S3
	R_1_	R_2_	R_3_	R_4_	R_5_	IC_50_ (μM)	IC_50_ (μM)
**5**	α-OH	α-Me	α-OH	α-OH	OH	>40	>40
**6**	α-OH	α-Me	α-OH	α-OH	OMe	19.83 ± 0.33	21.78 ± 1.13
**7**	=O	α-Me	=O	H	OH	32.88 ± 1.16	>40
**8**	=O	α-Me	=O	H	OMe	24.44 ± 0.34	27.14 ± 1.36
**9**	=O	α-Me	α-OH	α-OH	OH	>40	>40
**10**	=O	α-Me	α-OH	α-OH	OMe	21.77 ± 0.86	>40
**11**	=O	α-Me	α-OH	H	OH	32.41 ± 1.27	>40
**12**	=O	α-Me	α-OH	H	OMe	22.29 ± 0.19	29.00 ± 0.55
**13**	α-OH	α-Me, β-OH	β-OH	H	OH	>40	>40
**14**	α-OH	α-Me, β-OH	β-OH	H	OMe	>40	>40
**15**	α-OH	α-Me	=O	α-OH	OH	>40	>40
**16**	α-OH	α-Me	=O	α-OH	OMe	28.23 ± 0.01	27.26 ± 1.66
**17**	α-OCOH	α-Me	=O	α-OH	OH	>40	>40
**18**	α-OCOH	α-Me	=O	α-OH	OMe	20.95 ± 3.54	>40
**19**	α-OH	α-Me	=O	H	OH	>40	>40
**20**	α-OH	α-Me	=O	H	OMe	>40	>40
**21**	=O	α-Me	β-Me	H	OH	>40	>40
**22**	=O	α-Me	β-Me	H	OMe	14.48 ± 1.00	26.79 ± 0.99
**23**	=O	α-Me	H	H	OH	>40	>40
**24**	=O	α-Me	H	H	OMe	>40	>40
**25**	α-OH	α-Me	β-OH	H	OH	>40	>40
**26**	α-OH	α-Me	β-OH	H	OMe	27.32 ± 0.33	>40
**27**	α-OH	α-Me	H	α-OH	OH	>40	>40
**28**	α-OH	α-Me	H	α-OH	OMe	30.98 ± 2.47	31.84 ± 2.67
**Paclitaxel**						1168.50 ± 75.02	12.11 ± 2.04

**Table 3:**
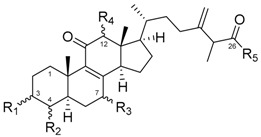
Collateral sensitivity evaluation of the effects of zhankuic acid-type compounds and their methyl esters on the cytotoxicity of paclitaxel.

No	Structure					Conc. (μM)	KBvin		HeLa S3	
							IC_50_ (nM)	RF ^1^	IC_50_ (nM)	RF ^1^
	Paclitaxel only					-	1168.50 ± 75.02	1.00	12.11 ± 2.04	1.00
	R_1_	R_2_	R_3_	R_4_	R_5_	-				
**5**	α-OH	α-Me	α-OH	α-OH	OH	10	1078.64 ± 77.01	1.08	7.96 ± 0.21 *	1.52
						20	972.99 ± 37.93 *	1.20	7.53 ± 0.47 *	1.61
**6**	α-OH	α-Me	α-OH	α-OH	OMe	5	89.06 ± 0.43 *	13.12	4.76 ± 0.01 *	2.55
						10	18.25 ± 4.97 *	64.02	4.13 ± 0.60 *	4.49
**7**	=O	α-Me	=O	H	OH	10	613.33 ± 2.78 *	1.91	7.94 ± 0.63 *	1.53
						20	249.51 ± 23.44 *	4.68	6.67 ± 0.27 *	1.82
**8**	=O	α-Me	=O	H	OMe	5	58.41 ± 6.46 *	20.01	4.73 ± 0.15 *	2.56
						10	1.75 ± 0.63 *	666.62	2.70 ± 1.32 *	4.49
**9**	=O	α-Me	α-OH	α-OH	OH	10	1075.33 ± 83.81	1.09	7.55 ± 0.63*	1.60
						20	974.82 ± 60.89	1.20	8.03 ± 0.12*	1.51
**10**	=O	α-Me	α-OH	α-OH	OMe	5	77.86 ± 7.25 *	15.01	4.43 ± 0.26 *	2.73
						10	52.92 ± 7.90 *	22.08	3.08 ± 1.20 *	3.93
**11**	=O	α-Me	α-OH	H	OH	10	730.30 ± 29.35 *	1.60	8.33 ± 0.16 *	1.45
						20	279.61 ± 7.84 *	4.18	6.51 ± 0.25 *	1.86
**12**	=O	α-Me	α-OH	H	OMe	5	171.88 ± 73.04 *	6.80	4.65 ± 0.08 *	2.60
						10	1.69 ± 0.17 *	692.59	2.61 ± 1.37 *	4.64
**13**	α-OH	α-Me, β-OH	β-OH	H	OH	10	987.07 ± 75.18	1.18	17.93 ± 2.86	0.68
						20	902.61 ± 7.57 *	1.29	9.11 ± 0.16	1.33
**14**	α-OH	α-Me, β-OH	β-OH	H	OMe	5	664.92 ± 38.20 *	1.76	5.09 ± 0.19 *	2.38
						10	293.99 ± 57.13 *	3.97	4.23 ± 1.42 *	2.86
**15**	α-OH	α-Me	=O	α-OH	OH	10	931.63 ± 24.81 *	1.25	15.44 ± 1.37	0.78
						20	722.92 ± 23.34 *	1.62	9.49 ± 0.62	1.28
**16**	α-OH	α-Me	=O	α-OH	OMe	5	63.35 ± 6.10 *	18.44	5.00 ± 0.06 *	2.42
						10	4.94 ± 3.31 *	236.38	3.36 ± 0.42 *	3.60
**17**	α-OCOH	α-Me	=O	α-OH	OH	10	729.20 ± 13.82 *	1.60	5.07 ± 0.75 *	2.39
						20	659.03 ± 28.57 *	1.77	4.99 ± 1.39 *	2.43
**18**	α-OCOH	α-Me	=O	α-OH	OMe	5	41.12 ± 9.34 *	28.41	4.01 ± 0.01 *	3.02
						10	26.08 ± 7.86 *	44.81	2.33 ± 0.78 *	5.21
**19**	α-OH	α-Me	=O	H	OH	10	682.65 ± 32.57 *	1.71	4.29 ±1.08 *	2.82
						20	564.84 ± 25.45 *	2.07	4.19 ± 1.40 *	2.89
**20**	α-OH	α-Me	=O	H	OMe	5	444.16 ± 44.85 *	2.63	4.89 ± 0.18 *	2.47
						10	103.47 ± 7.32 *	11.29	3.47 ± 0.32 *	3.49
**21**	=O	α-Me	β-Me	H	OH	10	769.34 ± 8.16 *	1.52	4.86 ± 0.51 *	2.49
						20	657.20 ± 30.86 *	1.78	4.60 ± 1.44 *	2.63
**22**	=O	α-Me	β-Me	H	OMe	5	17.80 ± 6.56 *	65.66	2.94 ± 0.22 *	4.12
						10	108.96 ± 15.25 *	10.72	0.51 ± 0.10 *	23.64
**23**	=O	α-Me	H	H	OH	10	669.22 ± 39.57 *	1.75	4.65 ± 0.55 *	2.60
						20	461.90 ± 46.61 *	2.53	4.26 ± 1.08 *	2.85
**24**	=O	α-Me	H	H	OMe	5	544.76 ± 24.01 *	2.14	4.62 ± 0.02 *	2.62
						10	136.03 ± 27.36 *	8.59	4.59 ± 0.48 *	2.64
**25**	α-OH	α-Me	β-OH	H	OH	10	852.31 ± 47.93 *	1.37	5.10 ± 0.36 *	2.37
						20	723.07 ± 6.95 *	1.62	5.07 ± 0.31 *	2.39
**26**	α-OH	α-Me	β-OH	H	OMe	5	6.33 ± 0.10 *	184.47	3.55 ± 0.29 *	3.41
						10	3.35 ± 1.00 *	348.80	3.38 ± 0.25 *	3.58
**27**	α-OH	α-Me	H	α-OH	OH	10	681.06 ± 12.91 *	1.72	5.42 ± 0.01 *	2.23
						20	558.44 ± 4.62 *	2.09	5.33 ± 0.04 *	2.27
**28**	α-OH	α-Me	H	α-OH	OMe	5	65.90 ± 5.37 *	17.73	4.68 ± 0.01 *	2.59
						10	3.93 ± 1.96 *	297.23	4.02 ± 0.01 *	3.01

^1^ RF: Reversal fold = IC_50_ for the paclitaxel-only group/IC_50_ for the co-treatment group. * denotes *p* < 0.05 as compared to IC_50_ for the paclitaxel-only group.

## Data Availability

Data are contained within the article and Supplementary Materials.

## Supplementary Materials

The following are available online at https://www.mdpi.com/article/10.3390/ph14090916/s1, Figures S1–S24: ^1^H NMR and ^13^C NMR spectrum of compound **6**, **8**, **10**, **12**, **14**, **16**, **18**, **20**, **22**, **26** and **28**.
