# Supplementary material for: Chemoreversal Agents from Taiwanofungus Genus and Their More Potent Methyl Derivatives Targeting Signal Transducer and Activator of Transcription 3 (STAT3) Phosphorylation
Source: Pharmaceuticals (Basel). 2021 Sep 10;14(9):916. doi: 10.3390/ph14090916 (PMC8464893; doi:10.3390/ph14090916)
Supplement: Supplementary file 1 [file pharmaceuticals-14-00916-s001.zip › pharmaceuticals-1347006-supplementary.pdf]

Chemoreversal agents from Taiwanofungus genus and their more potent methyl derivatives targeting STAT3 phosphorylation

Ko-Hua Yu [1] Chin-Chuan Hung [2,3,4], Tian-Shung Wu [1,5], Chin-Fu Chen [6], I-Ting Wu [2], Ping-Chung Kuo [1], Sio-Hong Lam [1] and Hsin-Yi Hung [1,*]

1 School of Pharmacy, College of Medicine, National Cheng Kung University, Tainan 701, Taiwan; s68071061@gs.ncku.edu.tw (K.-H.Y.); tswu@mail.ncku.edu.tw (T.-S.W.); z10502016@email.ncku.edu.tw (P.-C.K.); shlam@mail.ncku.edu.tw (S.-H.L.)

2 Department of Pharmacy, College of Pharmacy, China Medical University, Taichung 406, Taiwan; cchung@mail.cmu.edu.tw (C.-C.H.); 105015202@cmu.edu.tw (I.-T.W.)

3 Department of Pharmacy, China Medical University Hospital, Taichung 404, Taiwan

4 Department of Healthcare Administration, Asia University, Taichung 500, Taiwan

5 Department of Pharmacy, College of Pharmacy and Health Care, Tajen University, Pingtung, 907, Taiwan

[6] Department of Life Sciences, National Cheng Kung University, Tainan 701, Taiwan; chin-fu9999@gmail.com

* Correspondence: z10308005@email.ncku.edu.tw

Table of Content

Figure S1 ^{1}H NMR (500 MHz, $CDCl_3$) spectrum of methyl 3α,7α,12α-trihydroxy-4α-methylergosta-8,24(28)-dien-11-on-26-oate. (**6**)........ 5

Figure S2 ^{13}C NMR (125 MHz, $CDCl_3$) spectrum of methyl 3α,7α,12α-trihydroxy-4α-methylergosta-8,24(28)-dien-11-on-26-oate. (**6**)........ 6

Figure S3 ^{1}H NMR (500 MHz, $CDCl_3$) spectrum of zhankuic acid A methyl ester. (**8**)........ 7

Figure S4 ^{13}C NMR (125 MHz, $CDCl_3$) spectrum of zhankuic acid A methyl ester. (**8**)........ 8

Figure S5 ^{1}H NMR (500 MHz, $CDCl_3$) spectrum of methyl 7α, 12α-dihydroxy-3,11-dioxo-4α-methylergosta-8,24(28)-dien-26-oate. (**10**)........ 9

Figure S6 ^{13}C NMR (125 MHz, $CDCl_3$) spectrum of methyl 7α, 12α-dihydroxy-3,11-dioxo-4α-methylergosta-8,24(28)-dien-26-oate. (**10**)........ 10

Figure S7 ^{1}H NMR (500 MHz, $CDCl_3$) spectrum of methyl-7β-hydroxy-3,11-dioxo-4α-methylergosta-8,24(28)-dien-26-oate. (**12**)........ 11

Figure S8 ^{13}C NMR (125 MHz, $CDCl_3$) spectrum of methyl-7β-hydroxy-3,11-dioxo-4α-methylergosta-8,24(28)-dien-26-oate. (**12**)........ 12

Figure S9 ^{1}H NMR (500 MHz, acetone-d_6) spectrum of methyl 3α,4β,7β-trihydroxyergosta-8,24(28)-dien-11-on-26-oate (methyl antcamphorol D, **14**)........ 13

Figure S10 ^{13}C NMR (125 MHz, acetone-d_6) spectrum of methyl 3α,4β,7β-trihydroxyergosta-8,24(28)-dien-11-on-26-oate (methyl antcamphorol D, **14**)........ 14

Figure S11 ^{1}H NMR (500 MHz, $CDCl_3$) spectrum of methyl 3α,12α-dihydroxy 4α-methylergosta-8,24(28)-diene-7,11-dion-26-oate (methyl antcinate H, **16**). 15

Figure S12 ^{13}C NMR (125 MHz, $CDCl_3$) spectrum of methyl 3α,12α-dihydroxy 4α-methylergosta-8,24(28)-diene-7,11-dion-26-oate (methyl antcinate H, **16**) 16

Figure S13 ^{1}H NMR (500 MHz, $CDCl_3$) spectrum of zhankuic acid methyl ester C 3-O-formate. (**18**)........ 17

Figure S14 ^{13}C NMR (125 MHz, $CDCl_3$) spectrum of zhankuic acid methyl ester C 3-O-formate. (**18**)........ 18

Figure S15 ^{1}H NMR (500 MHz, $CDCl_3$) spectrum of methyl 3α-hydroxy-7,11-dioxo-4α-methylergosta-8,24(28)-dien-26-oate. (**20**) 19

Figure S16 ^{13}C NMR (125 MHz, $CDCl_3$) spectrum of methyl 3α-hydroxy-7,11-dioxo-4α-methylergosta-8,24(28)-dien-26-oate. (**20**) 20

Figure S17 ^{1}H NMR (500 MHz, $CDCl_3$) spectrum of methyl 7β-hydroxy-3,11-dioxo-4α-methylergosta-8,24(28)-dien-26-oate. (**22**) 21

Figure S18 ^{13}C NMR (125 MHz, $CDCl_3$) spectrum of methyl 7β-hydroxy-3,11-dioxo-4α-methylergosta-8,24(28)-dien-26-oate. (**22**) 22

Figure S19 ^{1}H NMR (500 MHz, $CDCl_3$) spectrum of methyl 4α-methylergosta-8,24(28)-diene-3,11-dion-26-oate. (**24**) 23

Figure S20 ^{13}C NMR (125 MHz, $CDCl_3$) spectrum of methyl 4α-methylergosta-8,24(28)-diene-3,11-dion-26-oate. (**24**) 24

Figure S21 ^{1}H NMR (500 MHz, $CDCl_3$) spectrum of methyl 3α,7β-dihydroxy-4α-methylergosta-8,24(28)-dien-11-on-26-oate. (**26**) 25

Figure S22 ^{13}C NMR (125 MHz, $CDCl_3$) spectrum of methyl 3α,7β-dihydroxy-4α-methylergosta-8,24(28)-dien-11-on-26-oate. (**26**) 26

Figure S23 ^{1}H NMR (500 MHz, $CDCl_3$) spectrum of methyl 3β,12β-dihydroxy-11-oxo-4β-methylergosta-8,24(28)-dien-26-oate (methyl antcin M). (**28**) 27

Figure S24 ^{13}C NMR (125 MHz, $CDCl_3$) spectrum of methyl 3β,12β-dihydroxy-11-oxo-4β-methylergosta-8,24(28)-dien-26-oate (methyl antcin M). (**28**) 28

Figure S25 References for known compounds 29

Figure S1 ¹H NMR (500 MHz, $CDCl_3$) spectrum of methyl 3α,7α,12α-trihydroxy-4α-methylergosta-8,24(28)-dien-11-on-26-oate. (6)

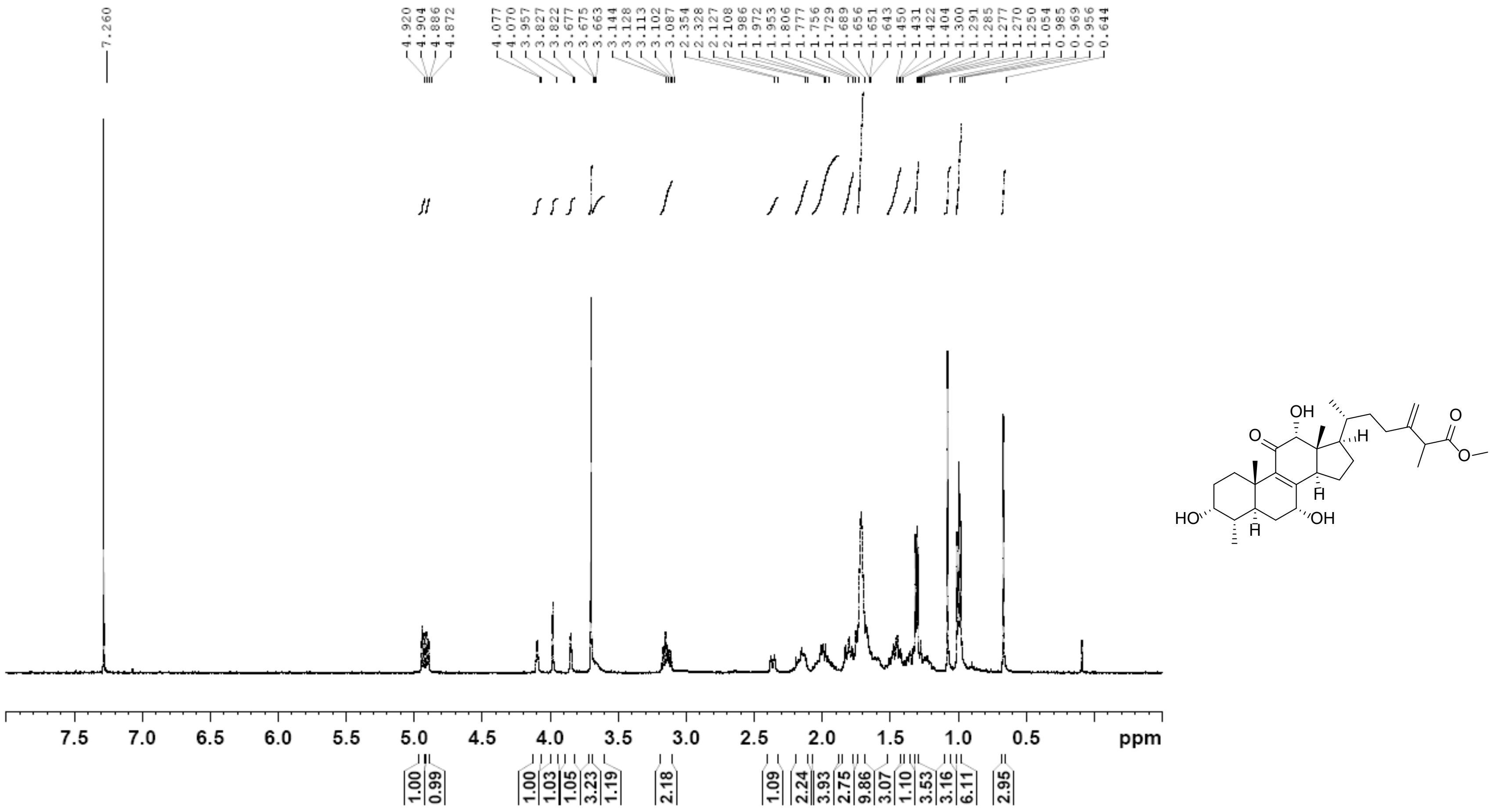

Figure S2 ^{13}C NMR (125 MHz, $CDCl_3$) spectrum of methyl 3α,7α,12α-trihydroxy-4α-methylergosta-8,24(28)-dien-11-on-26-oate. (6)

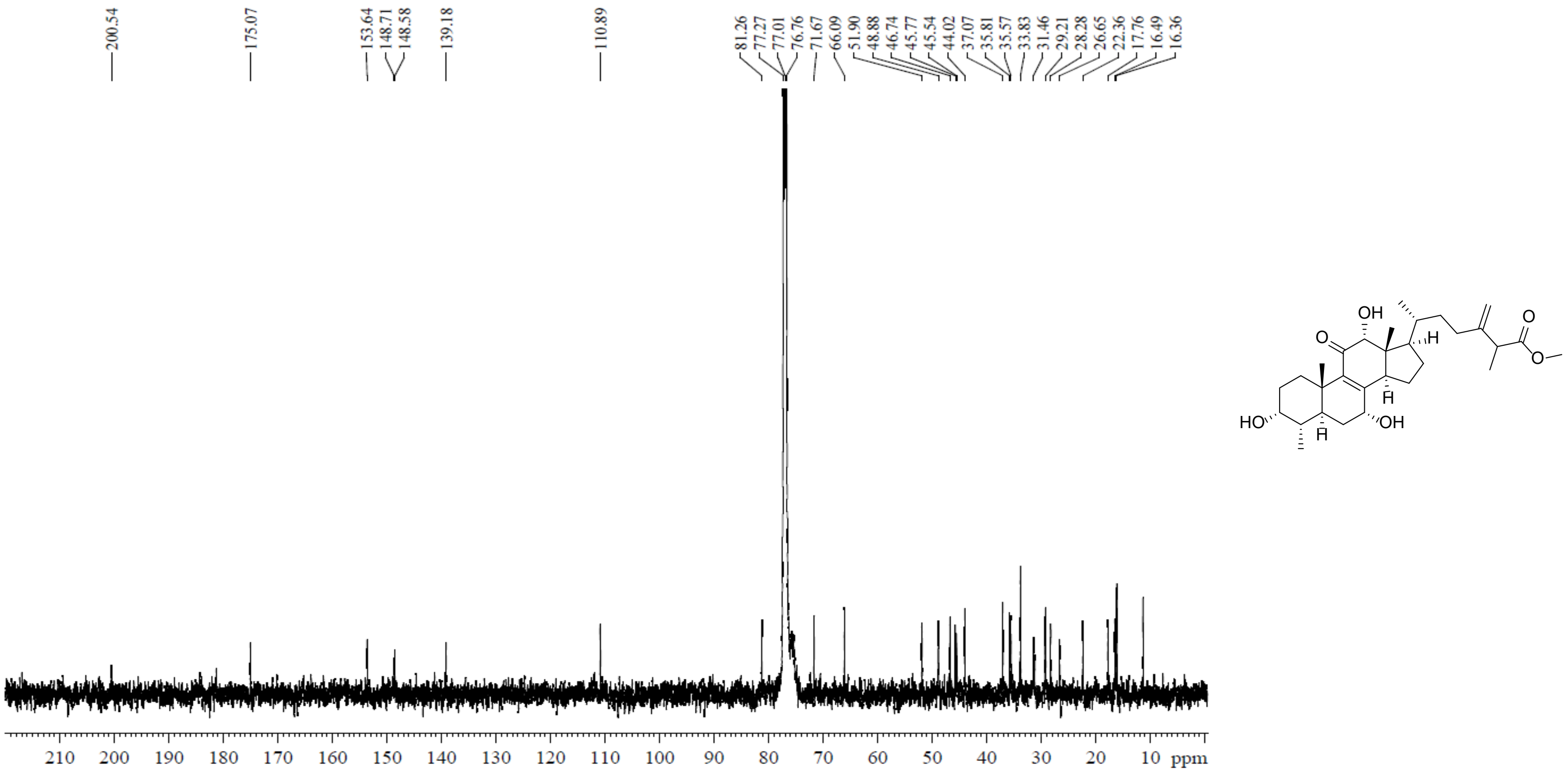

Figure S3 ^{1}H NMR (500 MHz, $CDCl_3$) spectrum of zhankuic acid A methyl ester. (8)

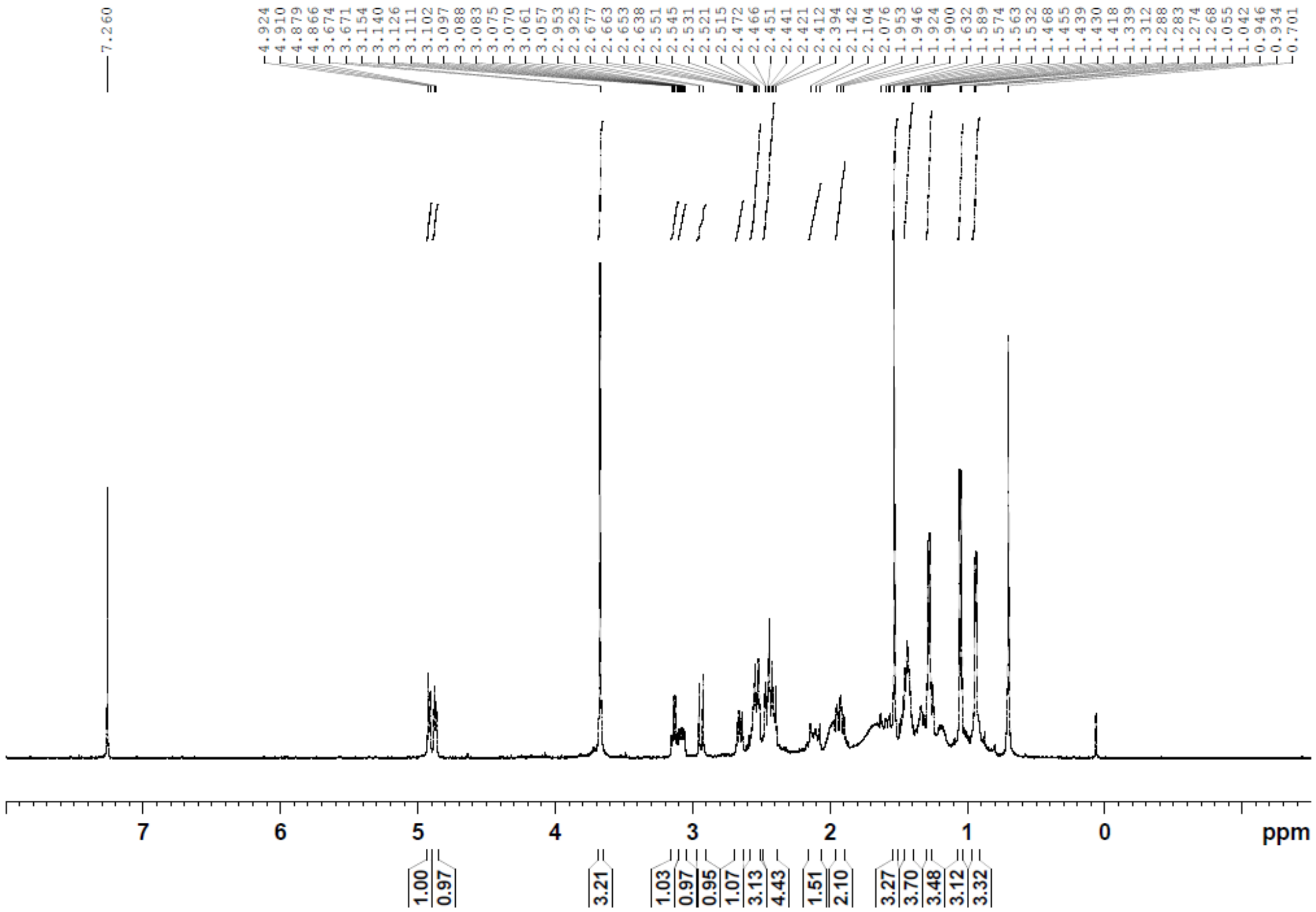

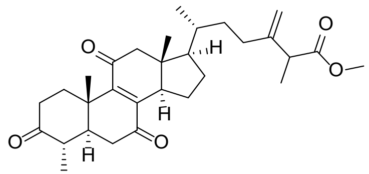

Figure S4 ^{13}C NMR (125 MHz, CDCl$_3$) spectrum of zhankuic acid A methyl ester. (8)

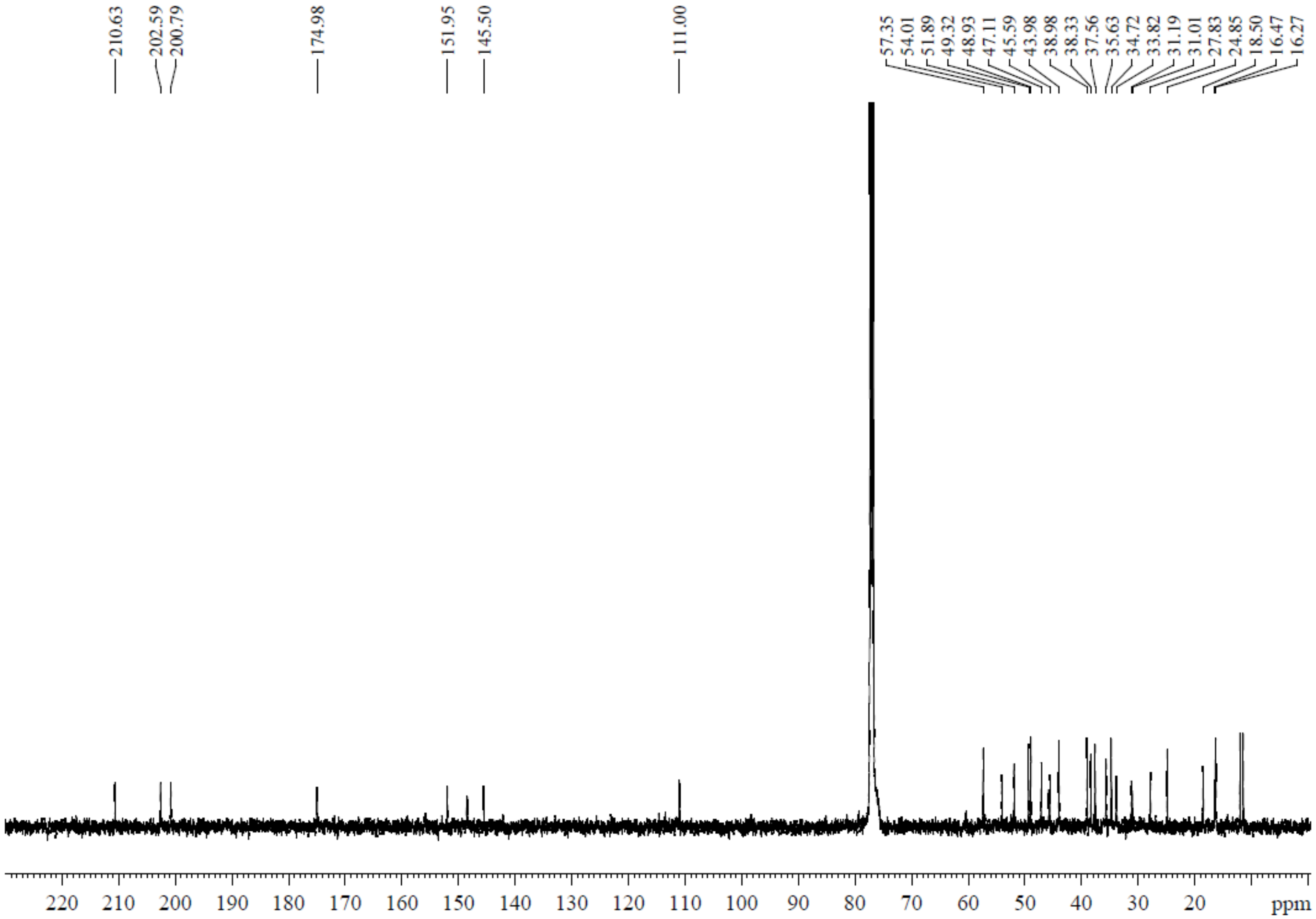

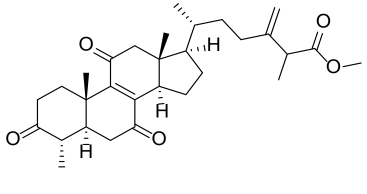

Figure S5 ^{1}H NMR (500 MHz, $CDCl_3$) spectrum of methyl 7α, 12α-dihydroxy-3,11-dioxo-4α-methylergosta-8,24(28)-dien-26-oate. (10)

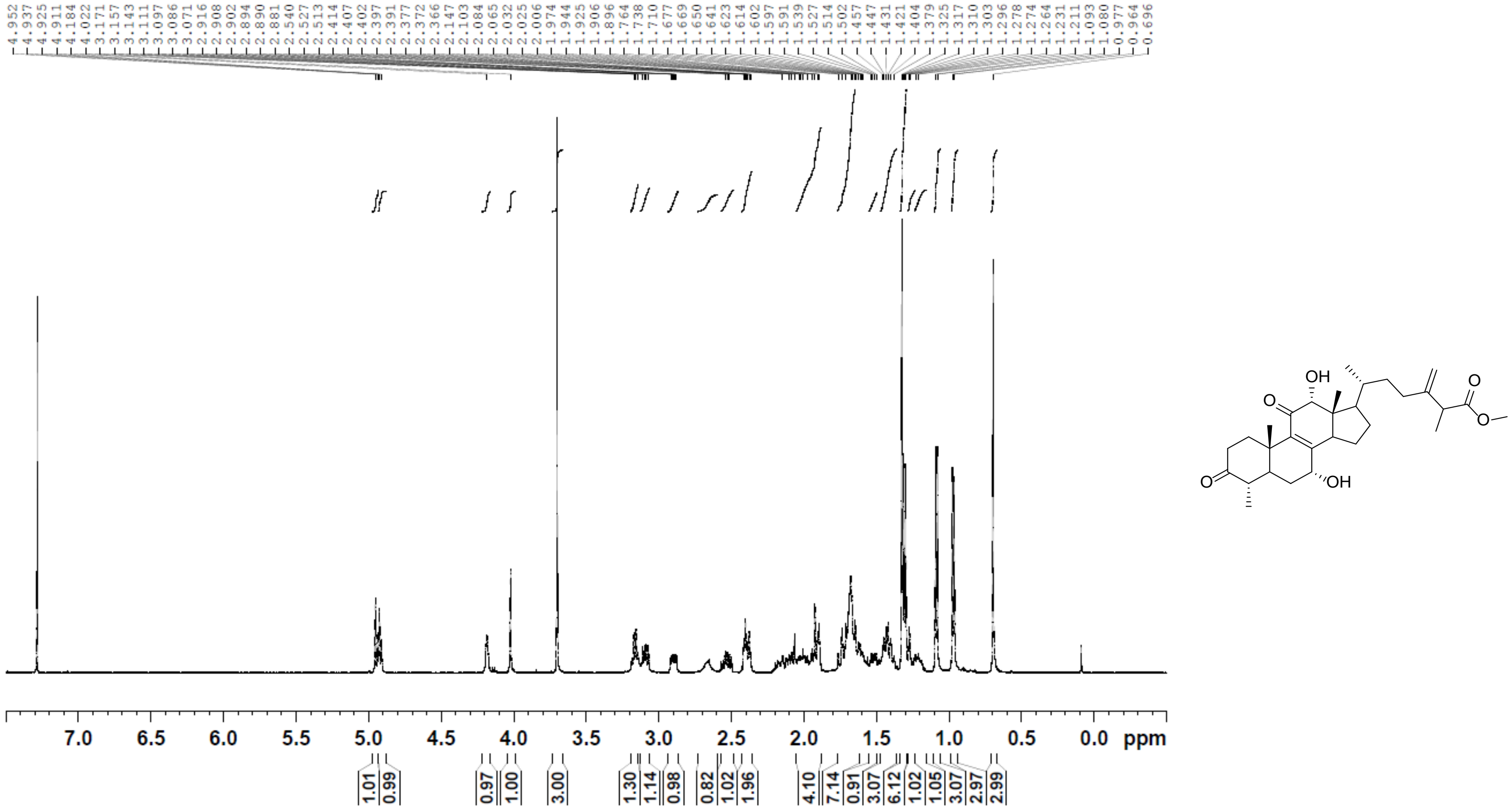

Figure S6 ^{13}C NMR (125 MHz, $CDCl_3$) spectrum of methyl 7α, 12α-dihydroxy-3,11-dioxo-4α-methylergosta-8,24(28)-dien-26-oate. (10)

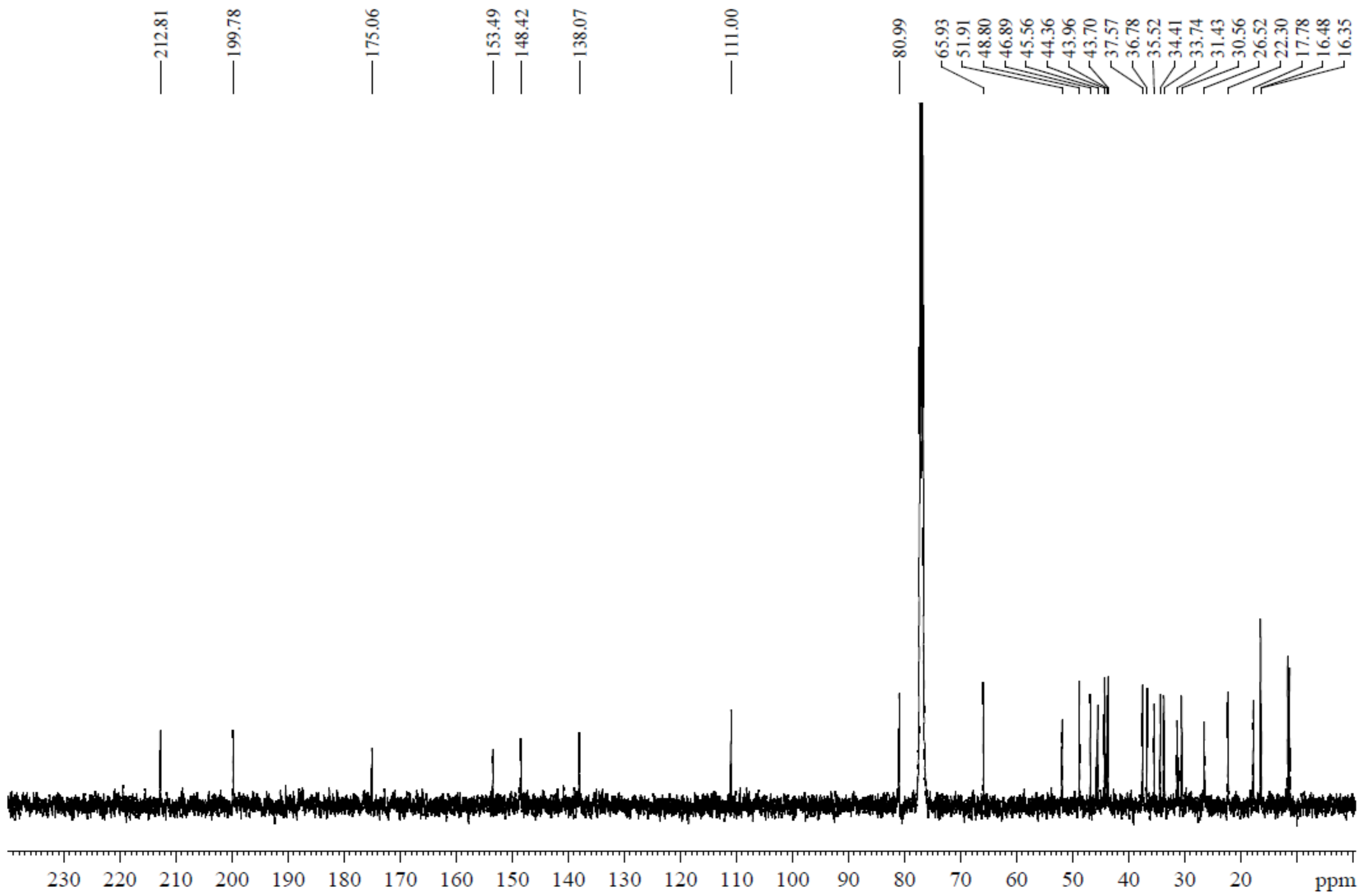

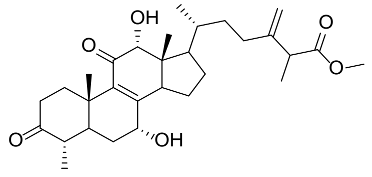

Figure S7 ^{1}H NMR (500 MHz, $CDCl_3$) spectrum of methyl-7β-hydroxy-3,11-dioxo-4α-methylergosta-8,24(28)-dien-26-oate. (12)

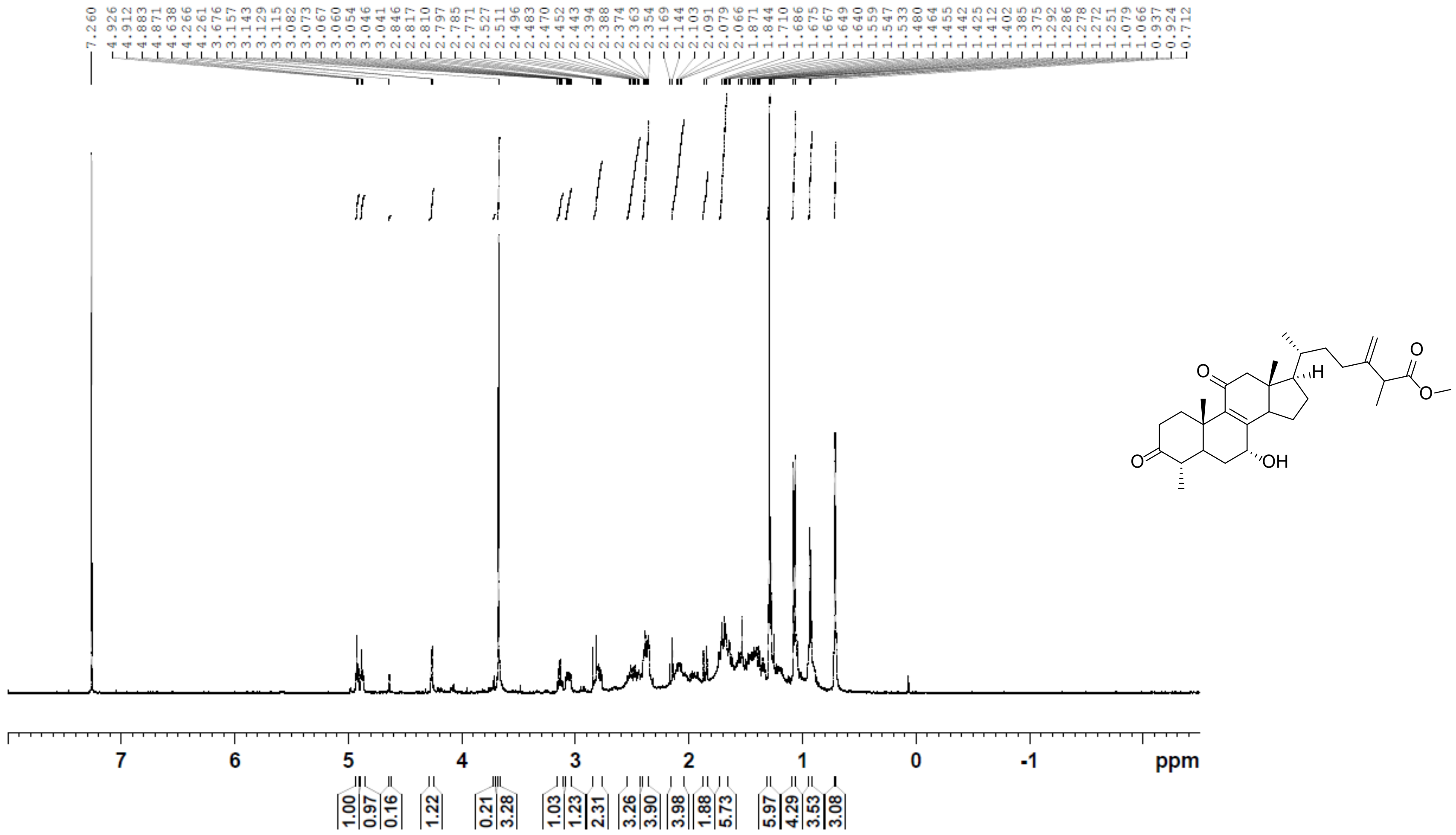

Figure S8 ^{13}C NMR (125 MHz, $CDCl_3$) spectrum of methyl-7β-hydroxy-3,11-dioxo-4α-methylergosta-8,24(28)-dien-26-oate. (12)

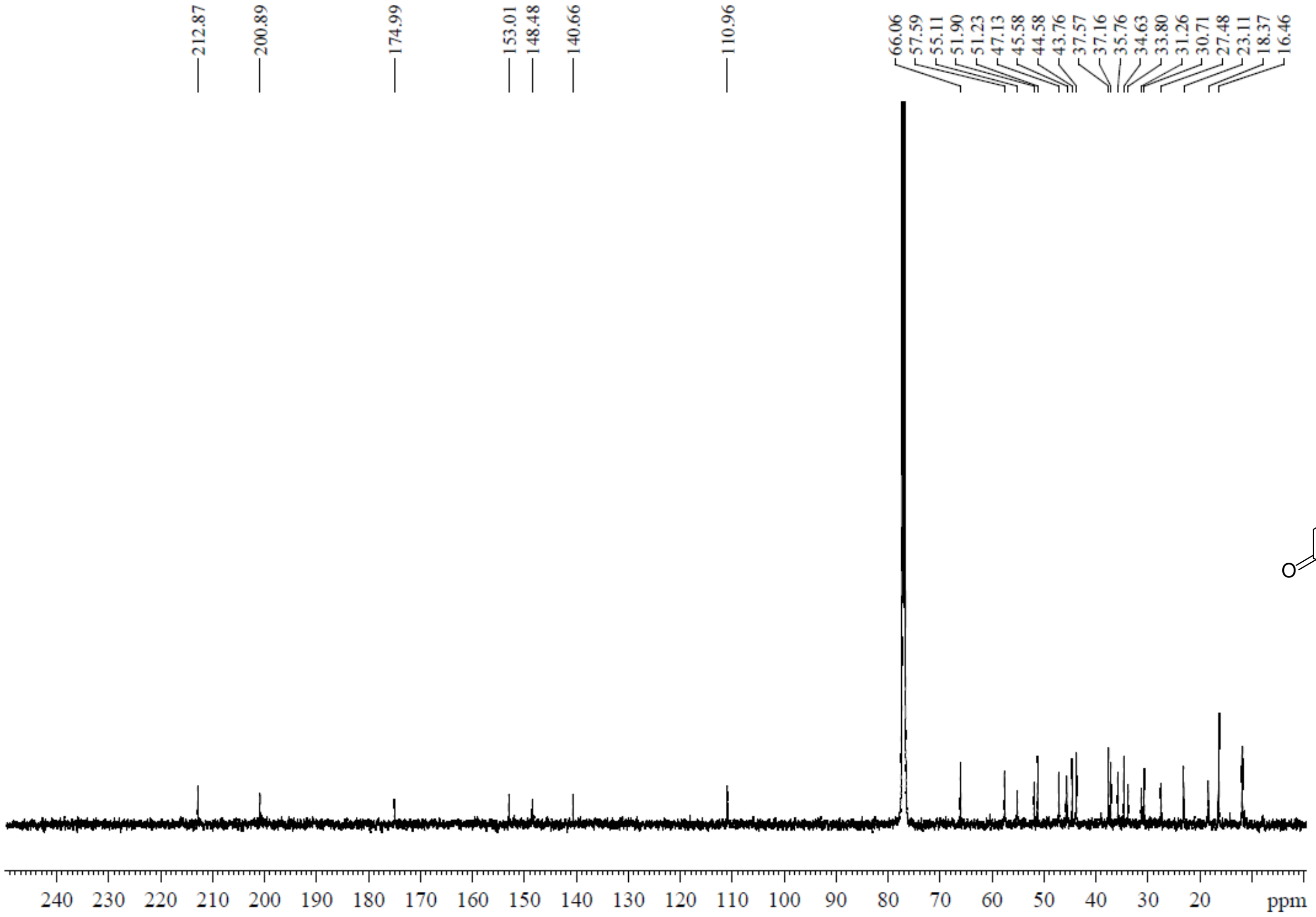

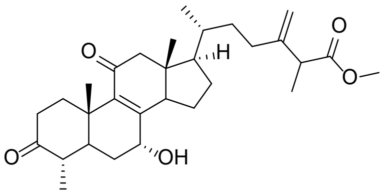

Figure S9 ¹H NMR (500 MHz, acetone-d$_6$) spectrum of methyl 3α,4β,7β-trihydroxyergosta-8,24(28)-dien-11-on-26-oate (methyl antcamphorol D, 14).

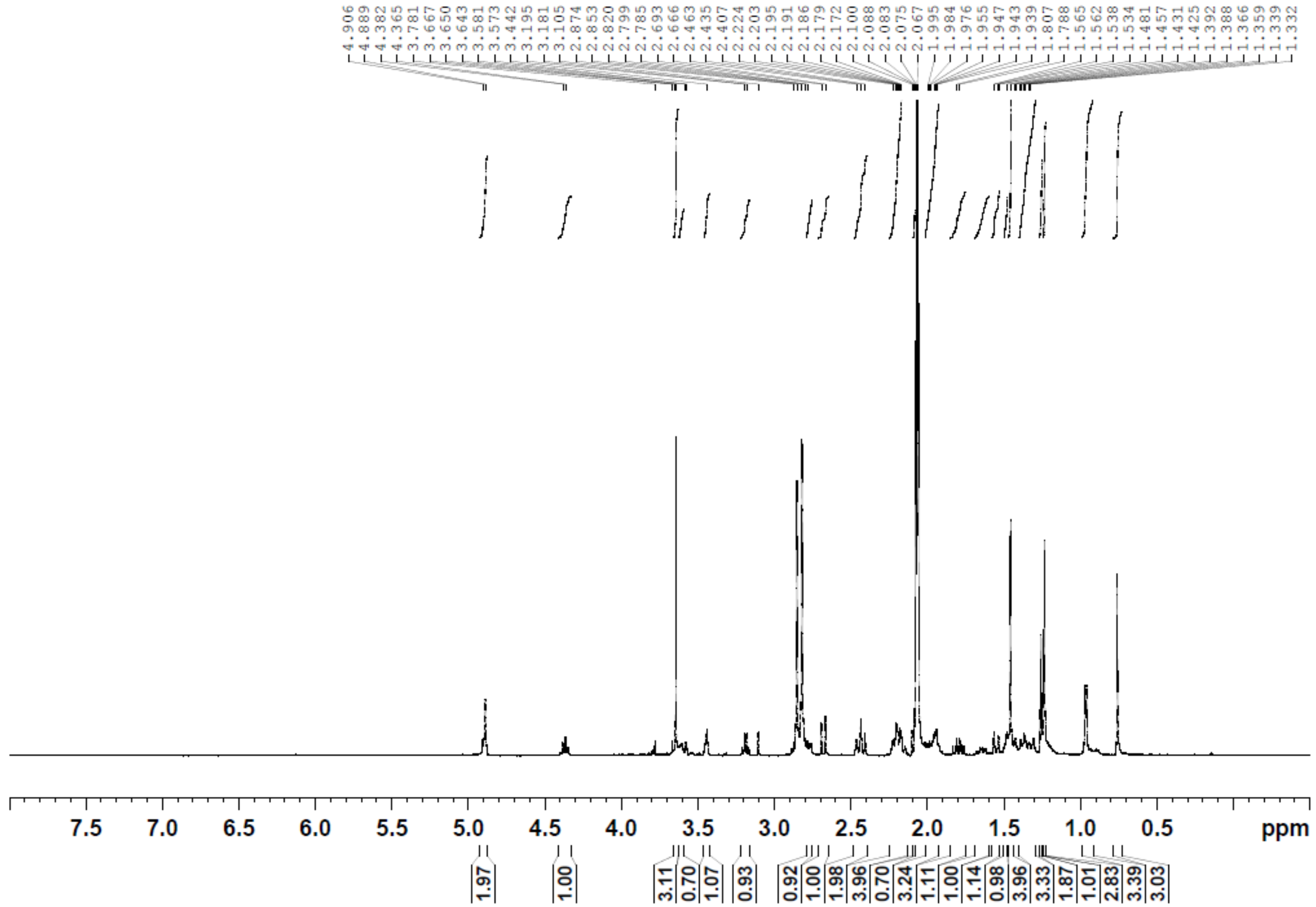

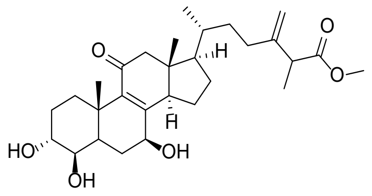

Figure S10 ^{13}C NMR (125 MHz, acetone-d_6) spectrum of methyl 3α,4β,7β-trihydroxyergosta-8,24(28)-dien-11-on-26-oate (methyl antcamphorol D, 14).

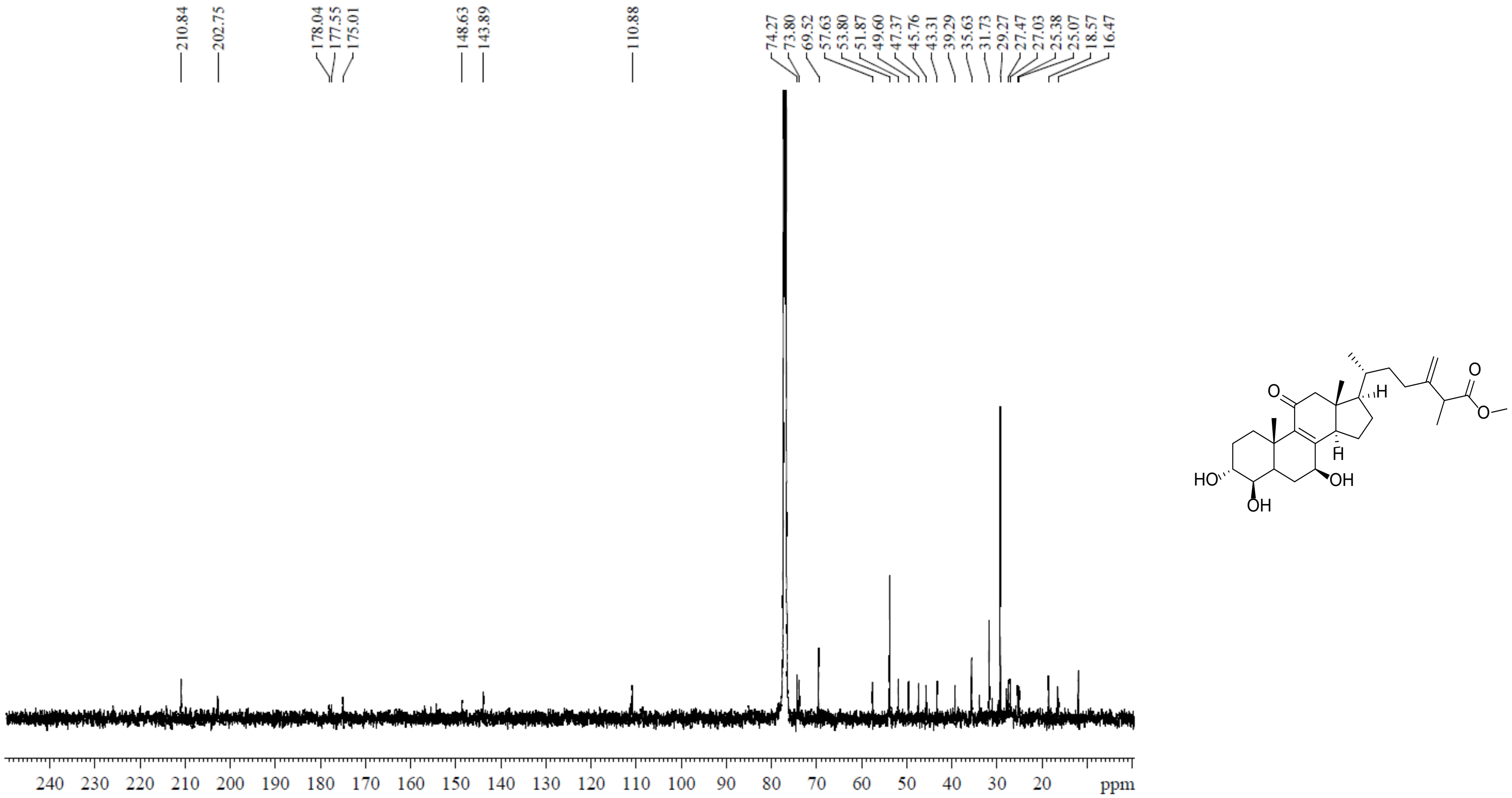

Figure S11 ^{1}H NMR (500 MHz, $CDCl_3$) spectrum of methyl 3α,12α-dihydroxy 4α-methylergosta-8,24(28)-diene-7,11-dion-26-oate (methyl antcinate H, 16).

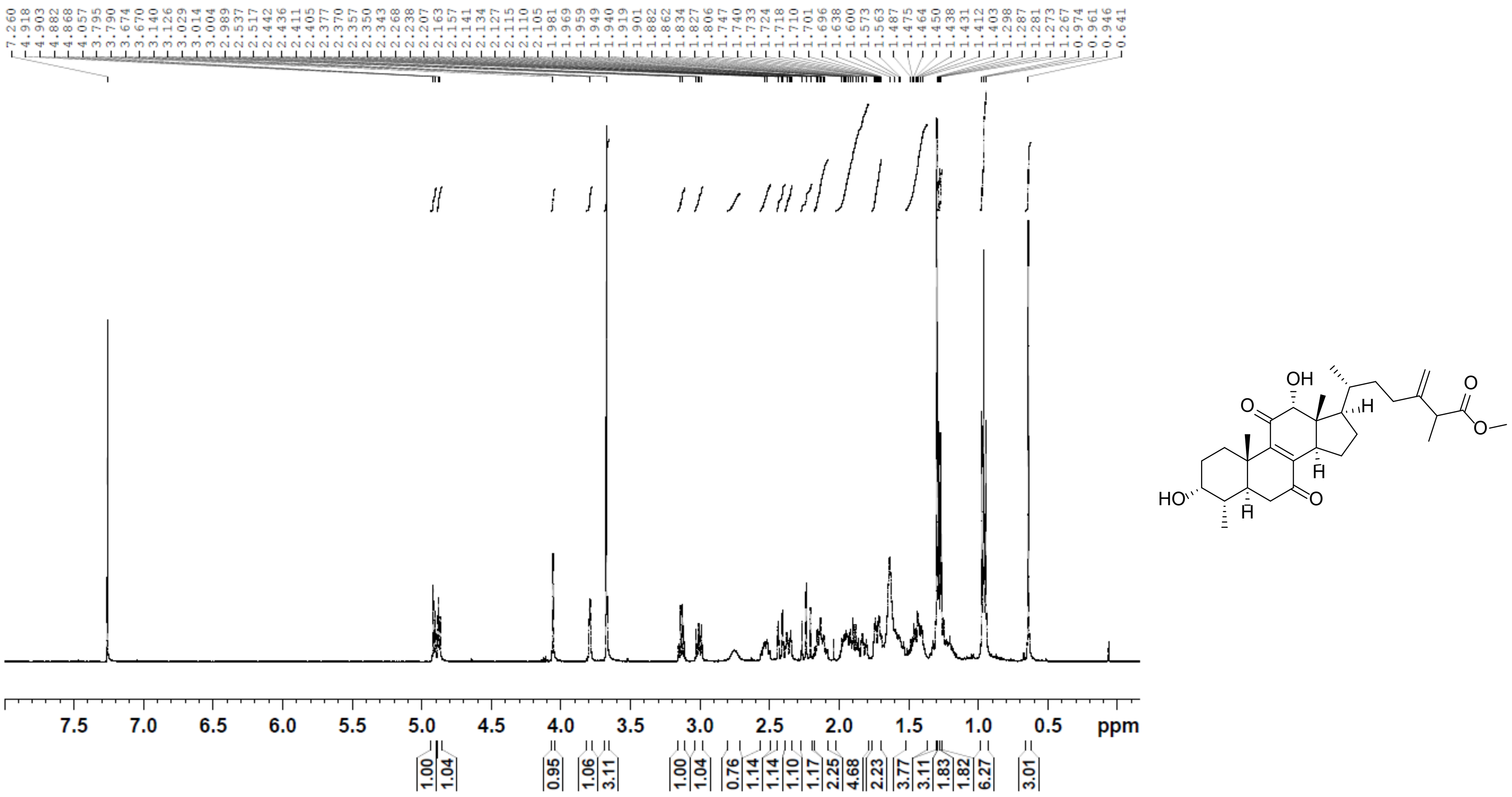

Figure S12 ^{13}C NMR (125 MHz, $CDCl_3$) spectrum of methyl 3α,12α-dihydroxy 4α-methylergosta-8,24(28)-diene-7,11-dion-26-oate (methyl antcinate H, 16)

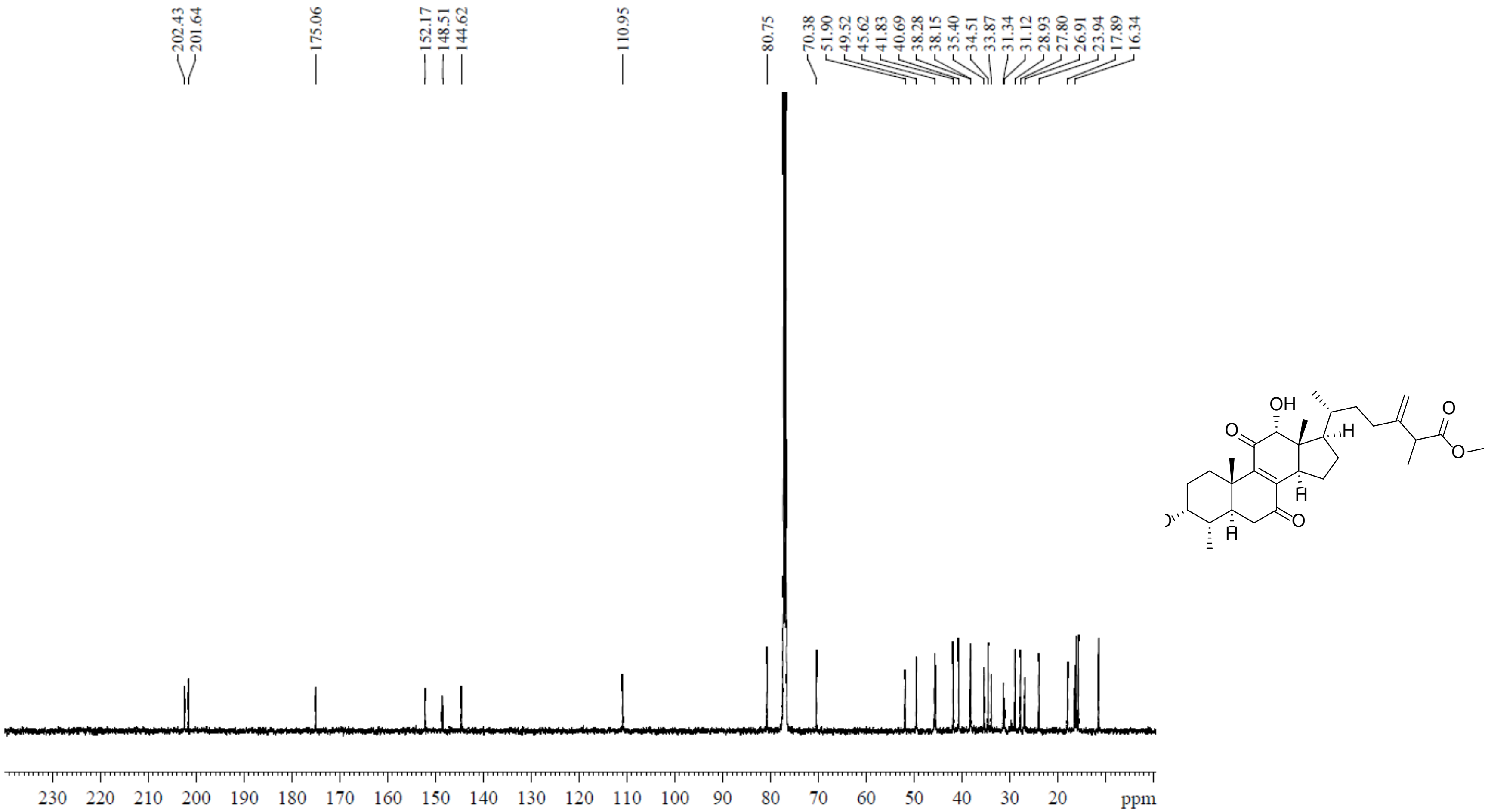

Figure S13 ^{1}H NMR (500 MHz, $CDCl_3$) spectrum of zhankuic acid methyl ester C 3-O-formate. (18)

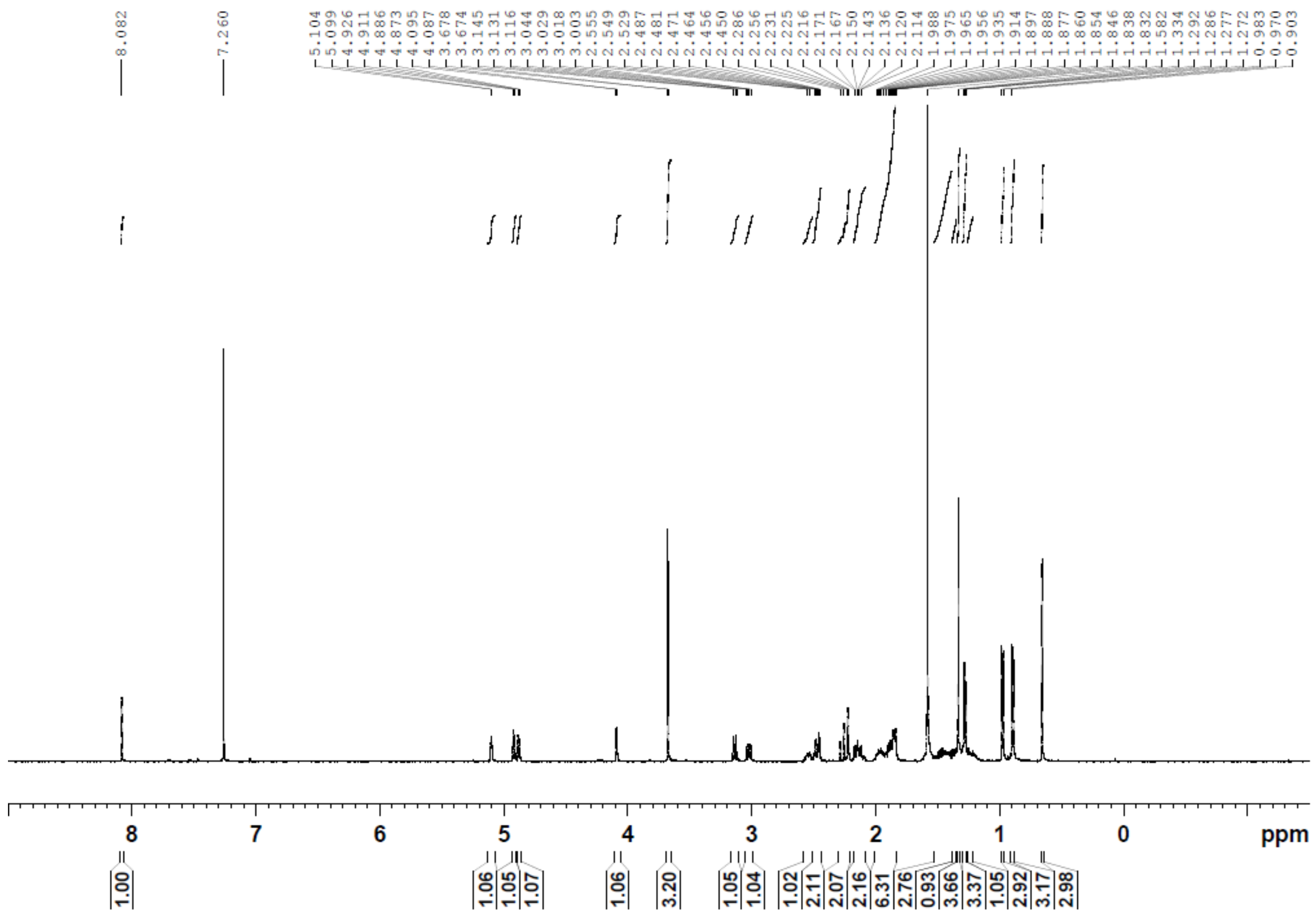

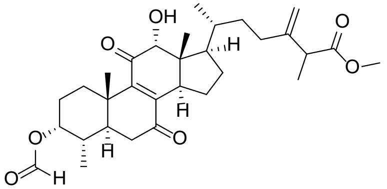

Figure S14 ^{13}C NMR (125 MHz, $CDCl_3$) spectrum of zhankuic acid methyl ester C 3-O-formate. (18)

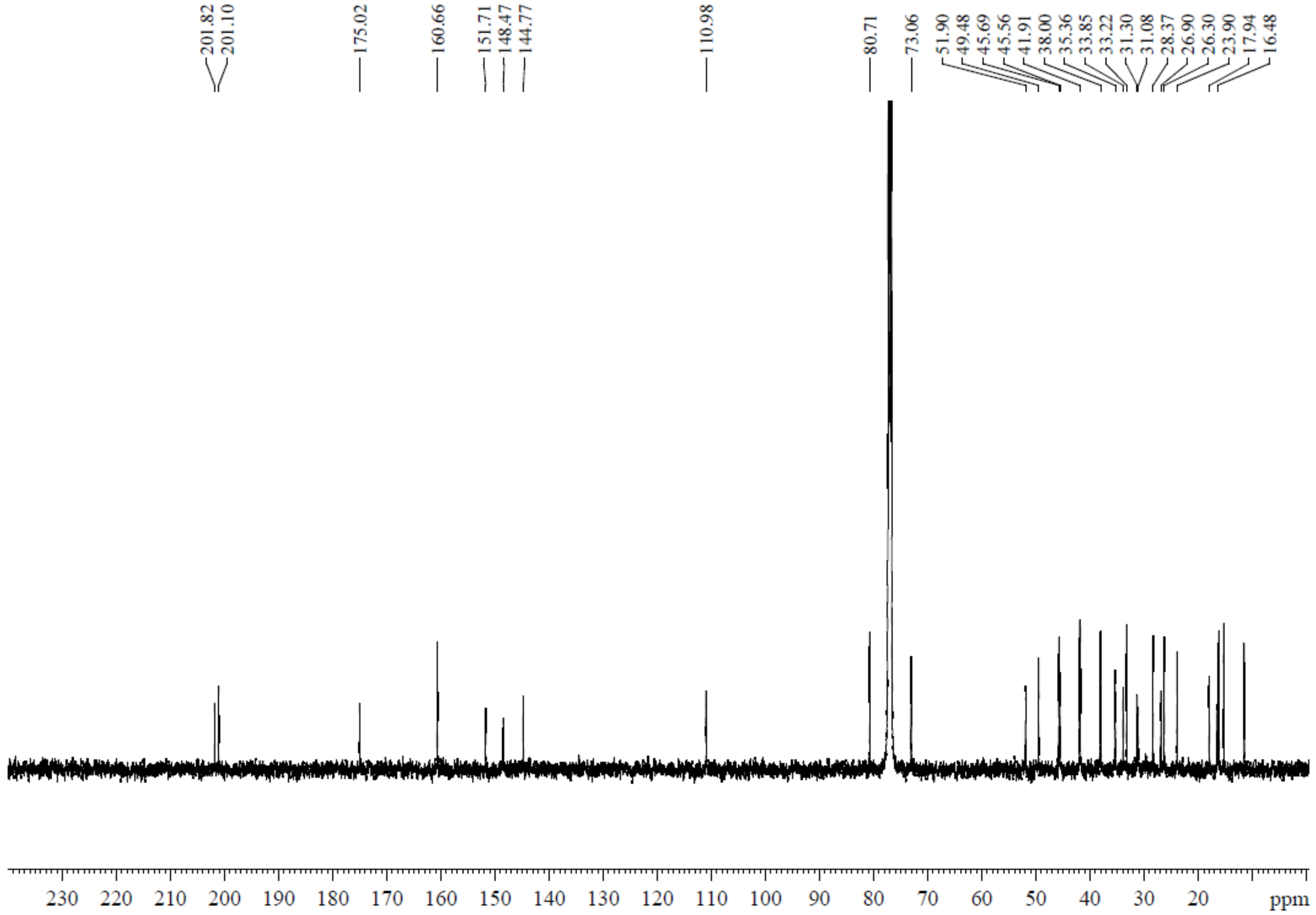

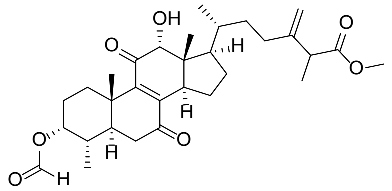

Figure S15 ^{1}H NMR (500 MHz, $CDCl_3$) spectrum of methyl 3α-hydroxy-7,11-dioxo-4α-methylergosta-8,24(28)-dien-26-oate. (20)

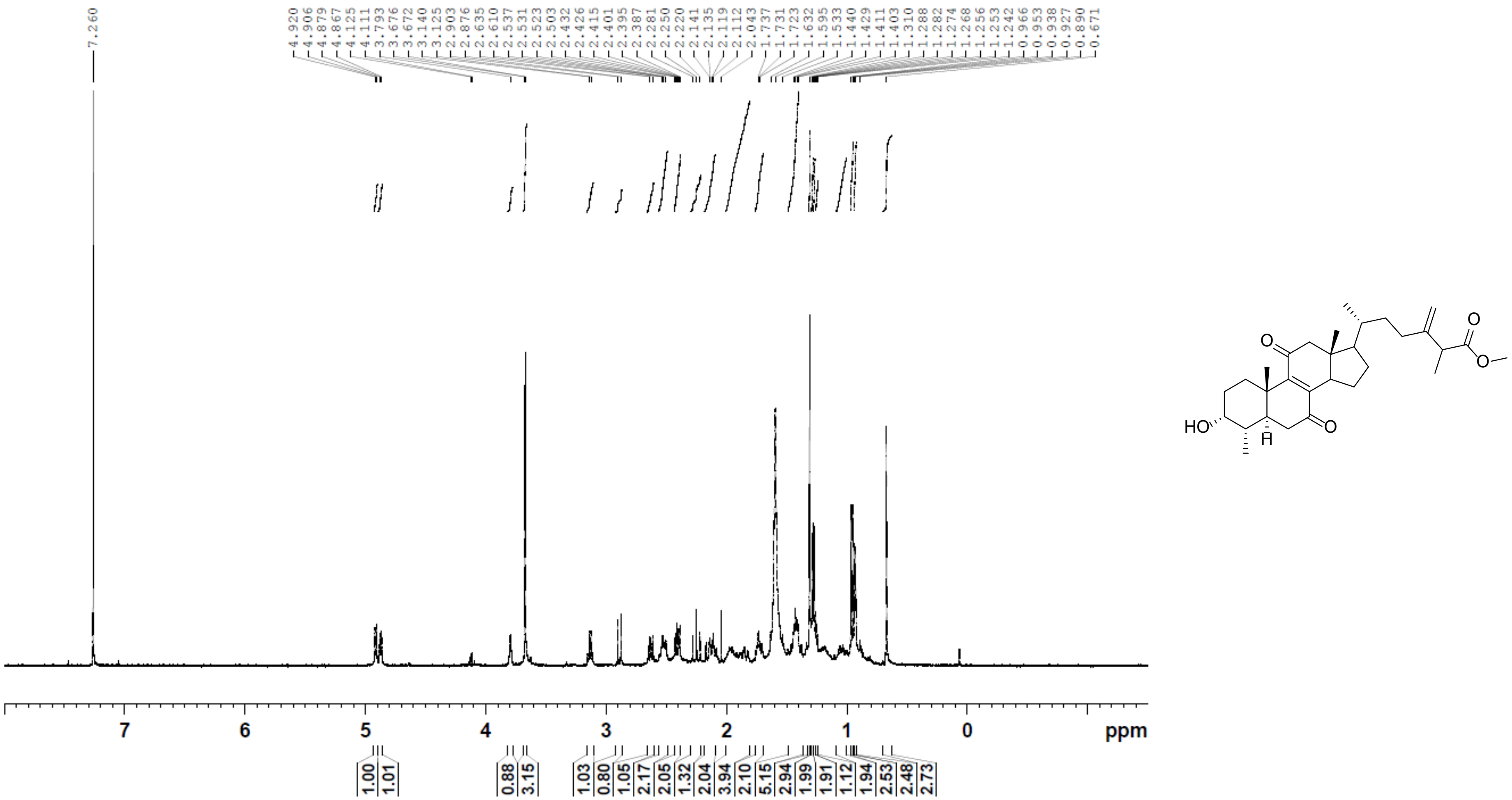

Figure S16 ^{13}C NMR (125 MHz, CDCl$_3$) spectrum of methyl 3α-hydroxy-7,11-dioxo-4α-methylergosta-8,24(28)-dien-26-oate. (20)

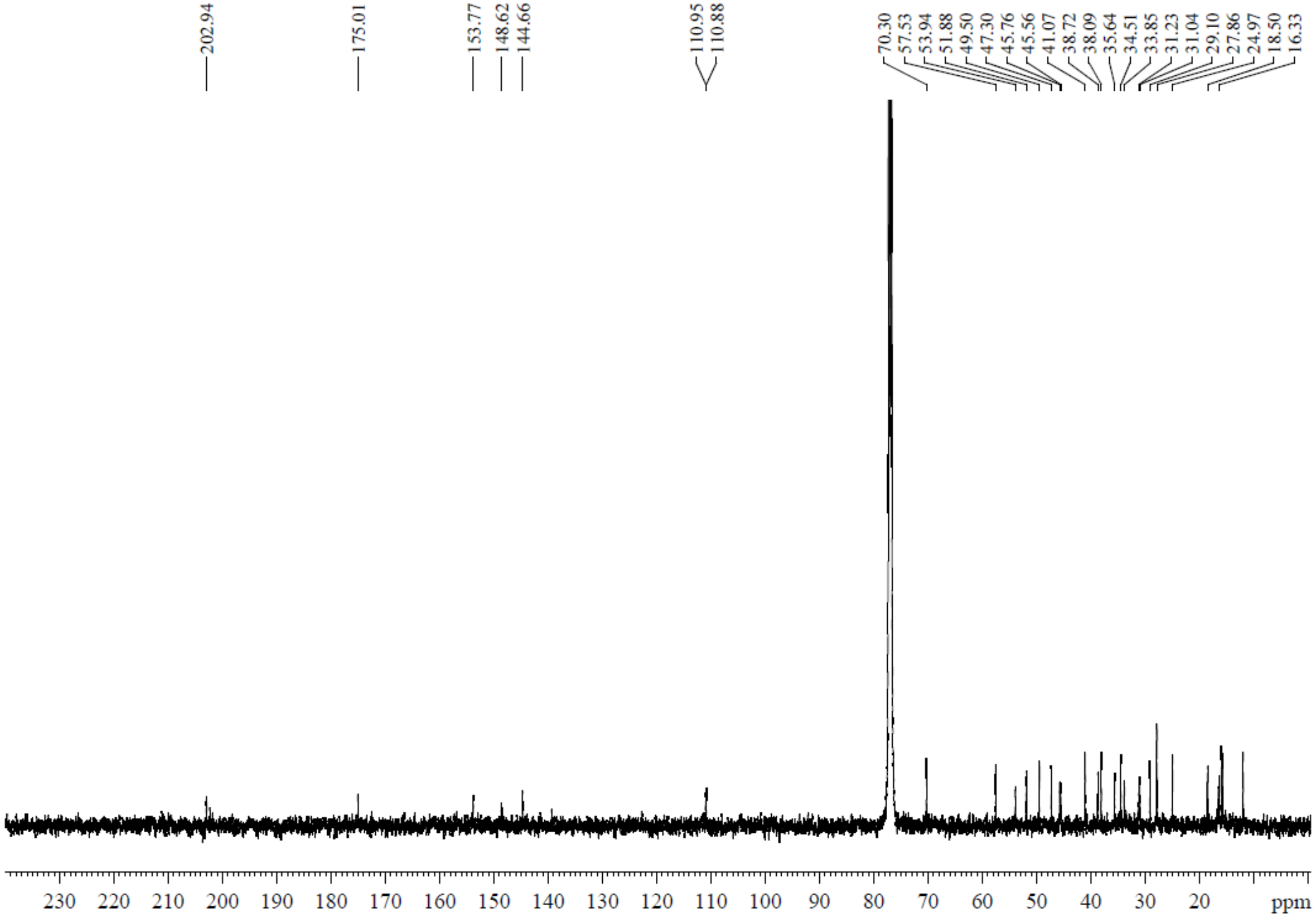

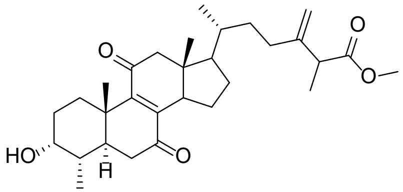

Figure S17 ^{1}H NMR (500 MHz, $CDCl_3$) spectrum of methyl 7β-hydroxy-3,11-dioxo-4α-methylergosta-8,24(28)-dien-26-oate. (22)

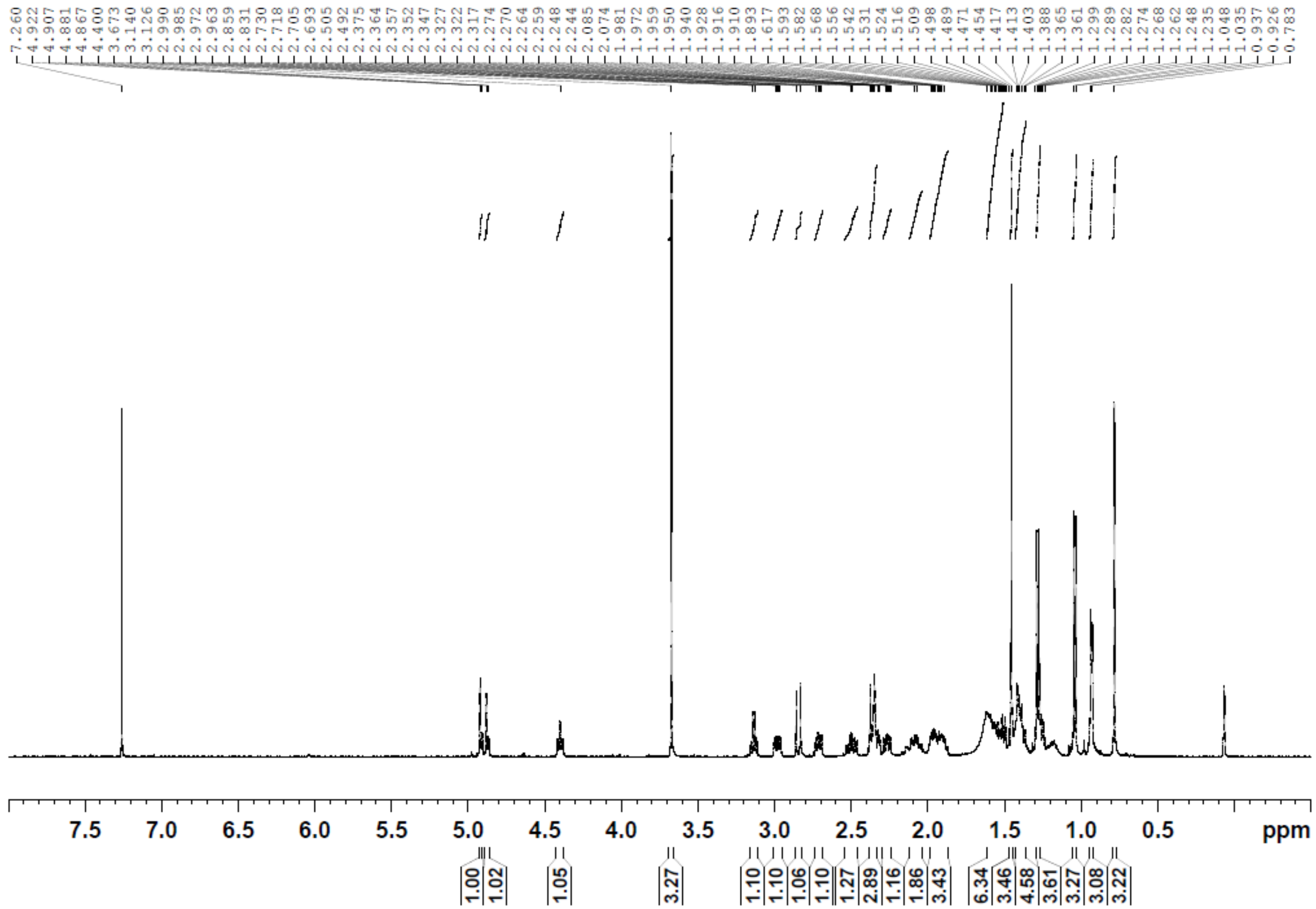

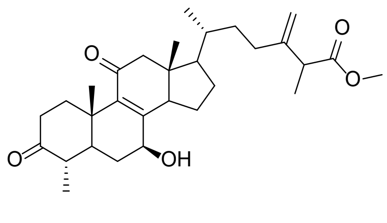

Figure S18 ^{13}C NMR (125 MHz, CDCl$_3$) spectrum of methyl 7β-hydroxy-3,11-dioxo-4α-methylergosta-8,24(28)-dien-26-oate. (22)

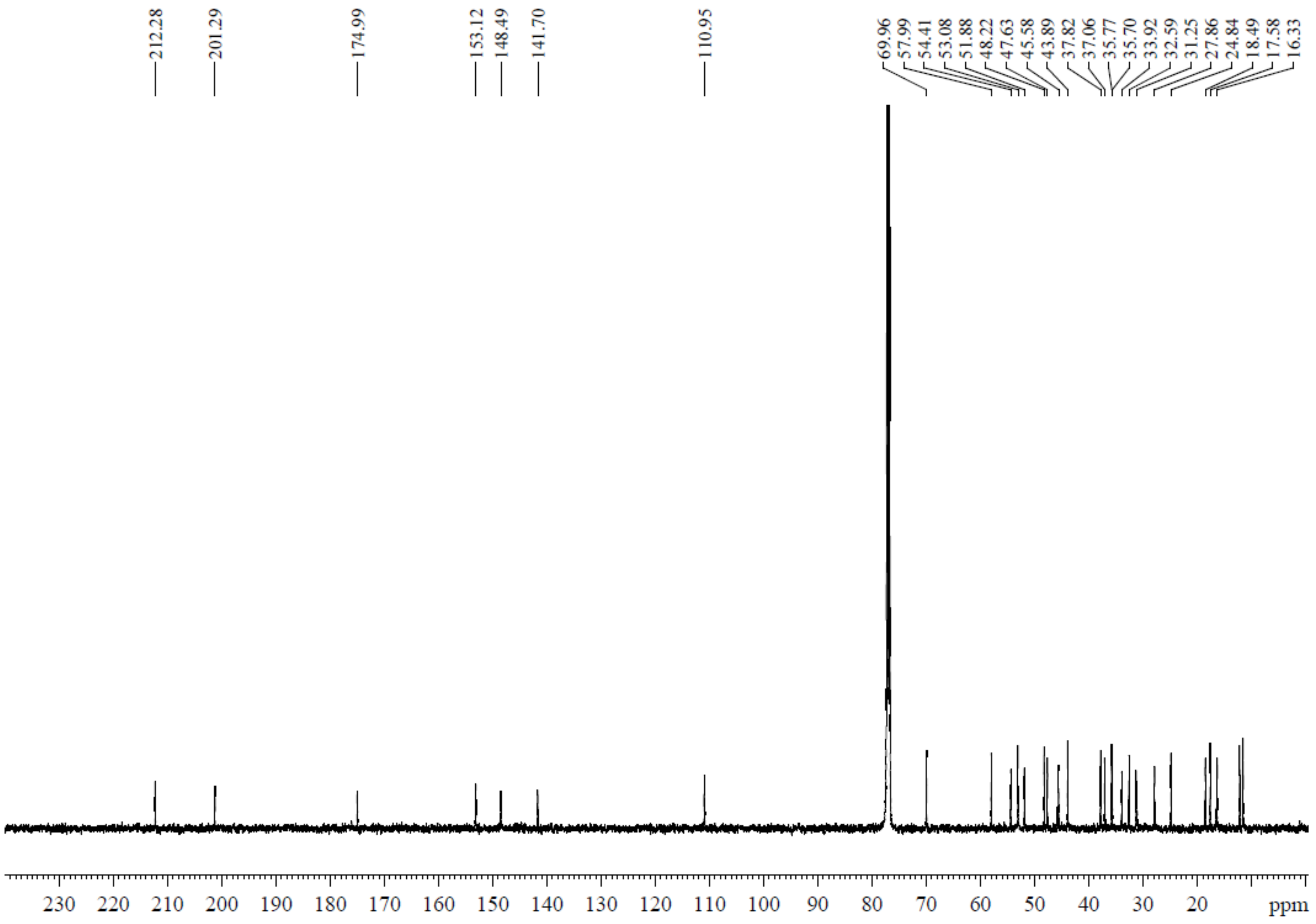

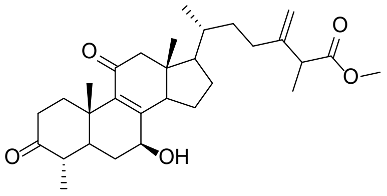

Figure S19 ¹H NMR (500 MHz, $CDCl_3$) spectrum of methyl 4α-methylergosta-8,24(28)-diene-3,11-dion-26-oate. (24)

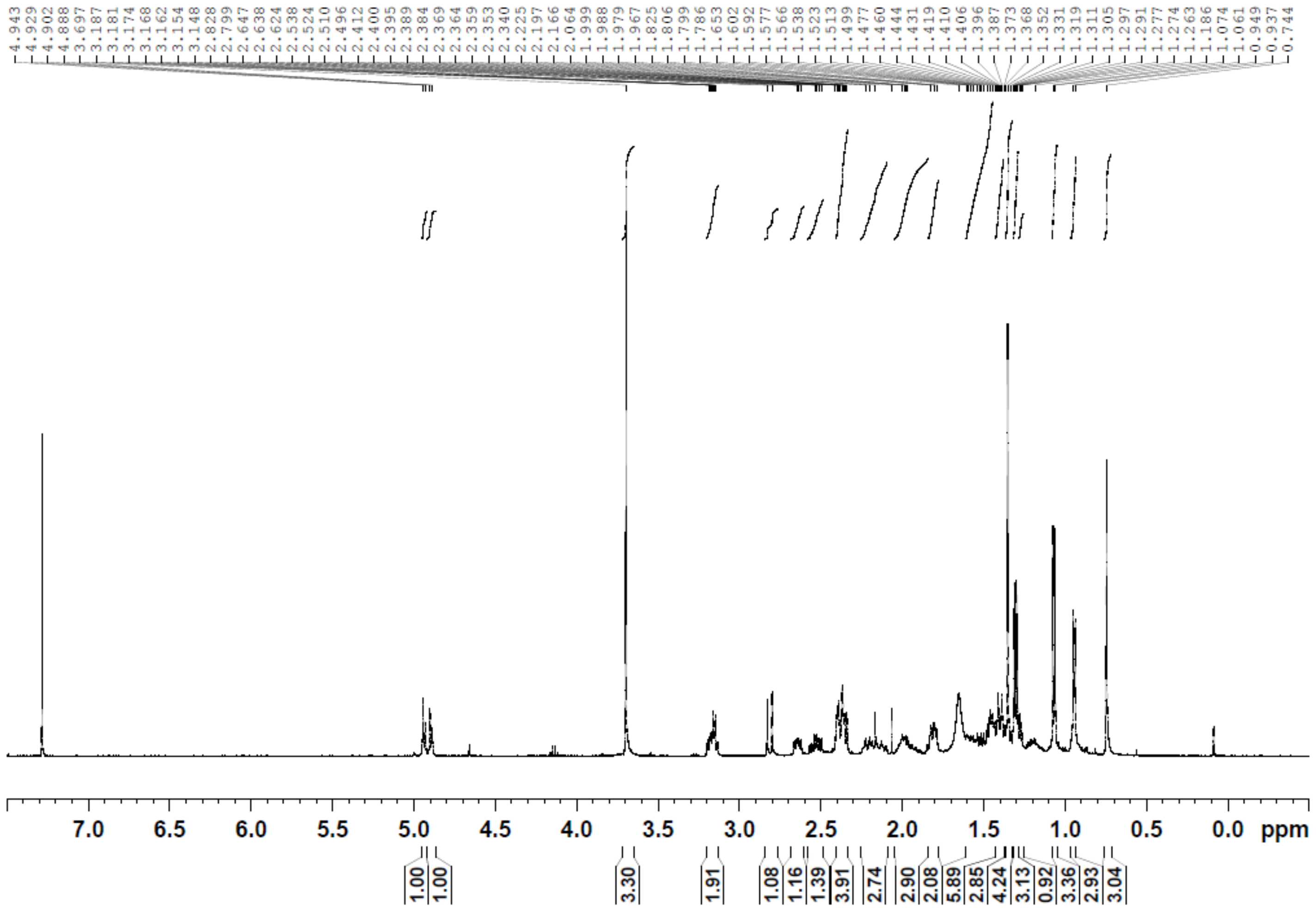

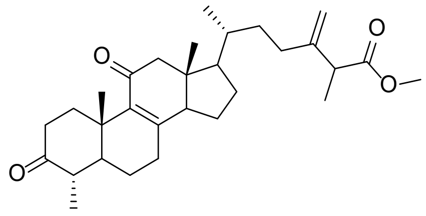

Figure S20 ^{13}C NMR (125 MHz, $CDCl_3$) spectrum of methyl 4α-methylergosta-8,24(28)-diene-3,11-dion-26-oate. (24)

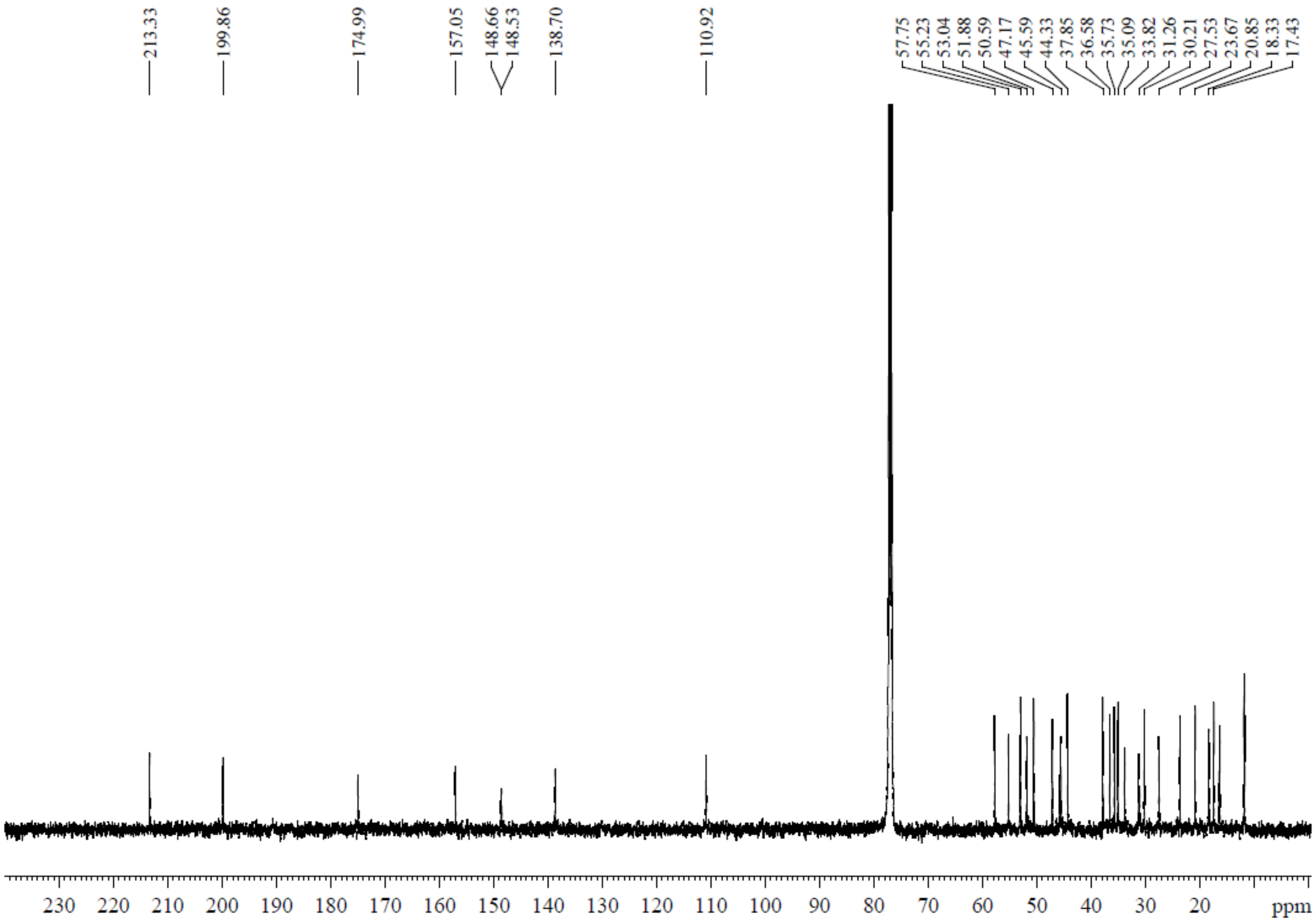

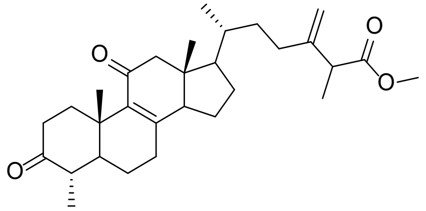

Figure S21 ¹H NMR (500 MHz, CDCl₃) spectrum of methyl 3α,7β-dihydroxy-4α-methylergosta-8,24(28)-dien-11-on-26-oate. (26)

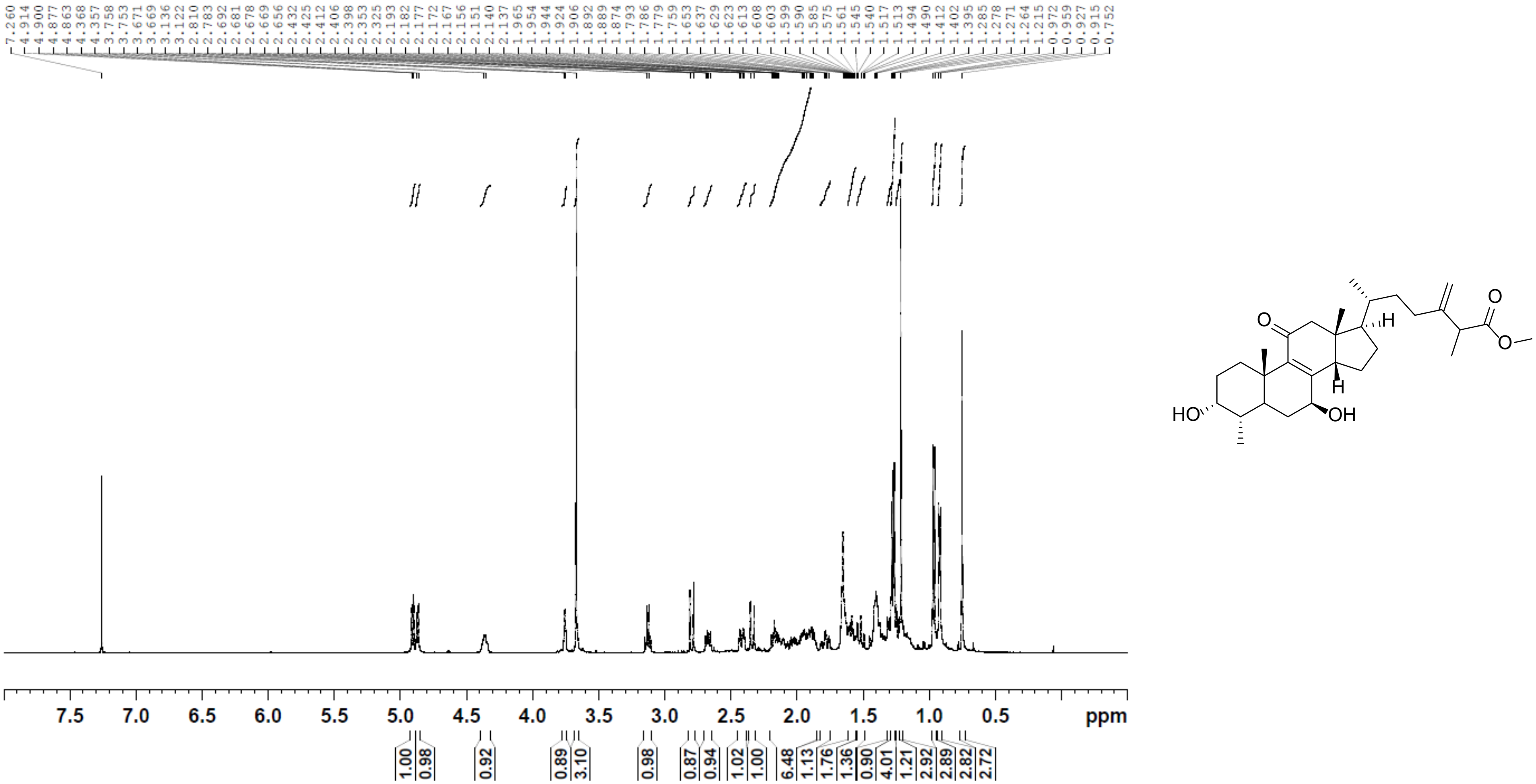

Figure S22 ^{13}**C NMR (125 MHz, CDCl$_3$) spectrum of methyl 3α,7β-dihydroxy-4α-methylergosta-8,24(28)-dien-11-on-26-oate. (26)**

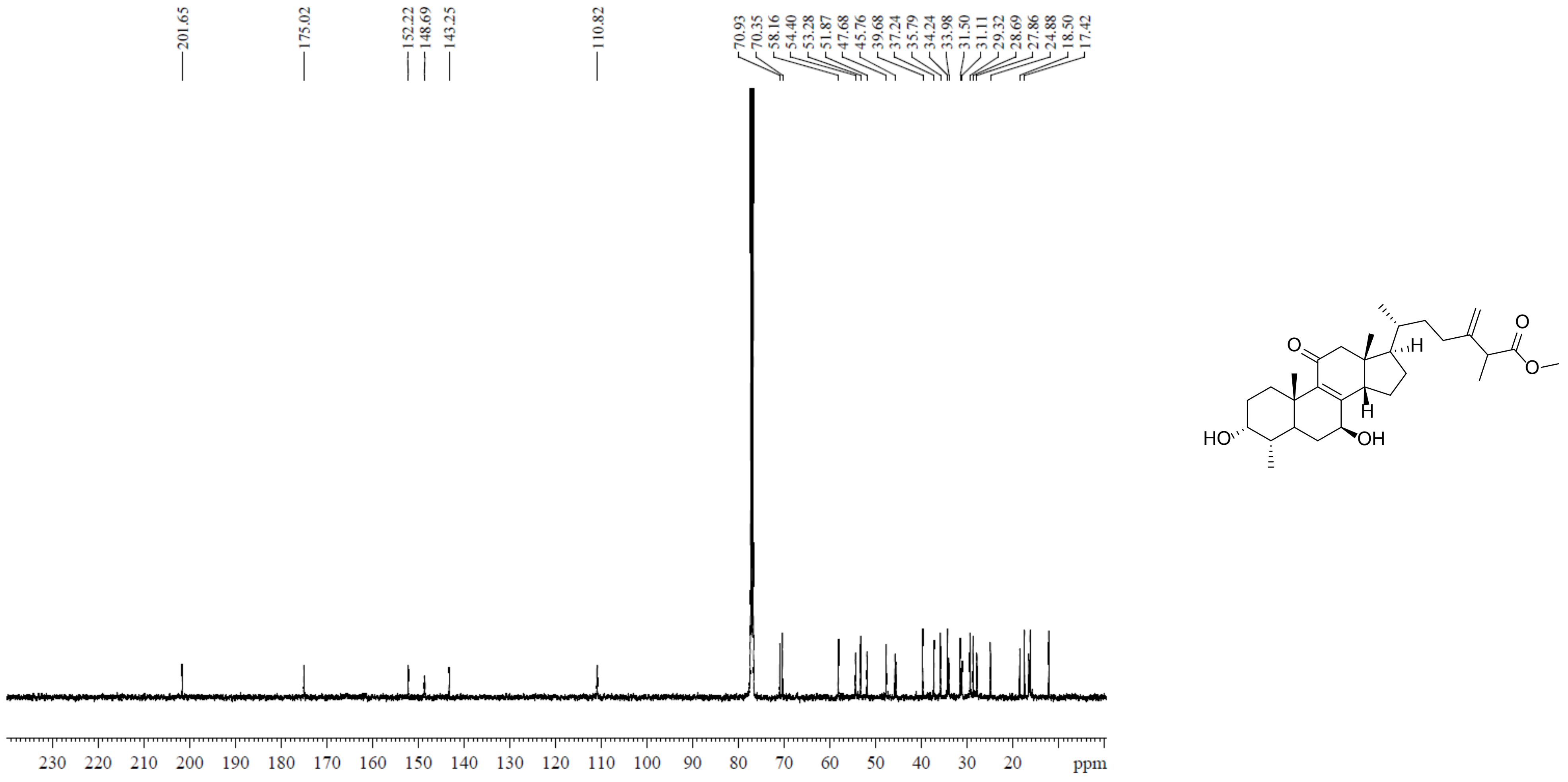

Figure S23 ^{1}H NMR (500 MHz, $CDCl_3$) spectrum of methyl 3β,12β-dihydroxy-11-oxo-4β-methylergosta-8,24(28)-dien-26-oate (methyl antcin M). (28)

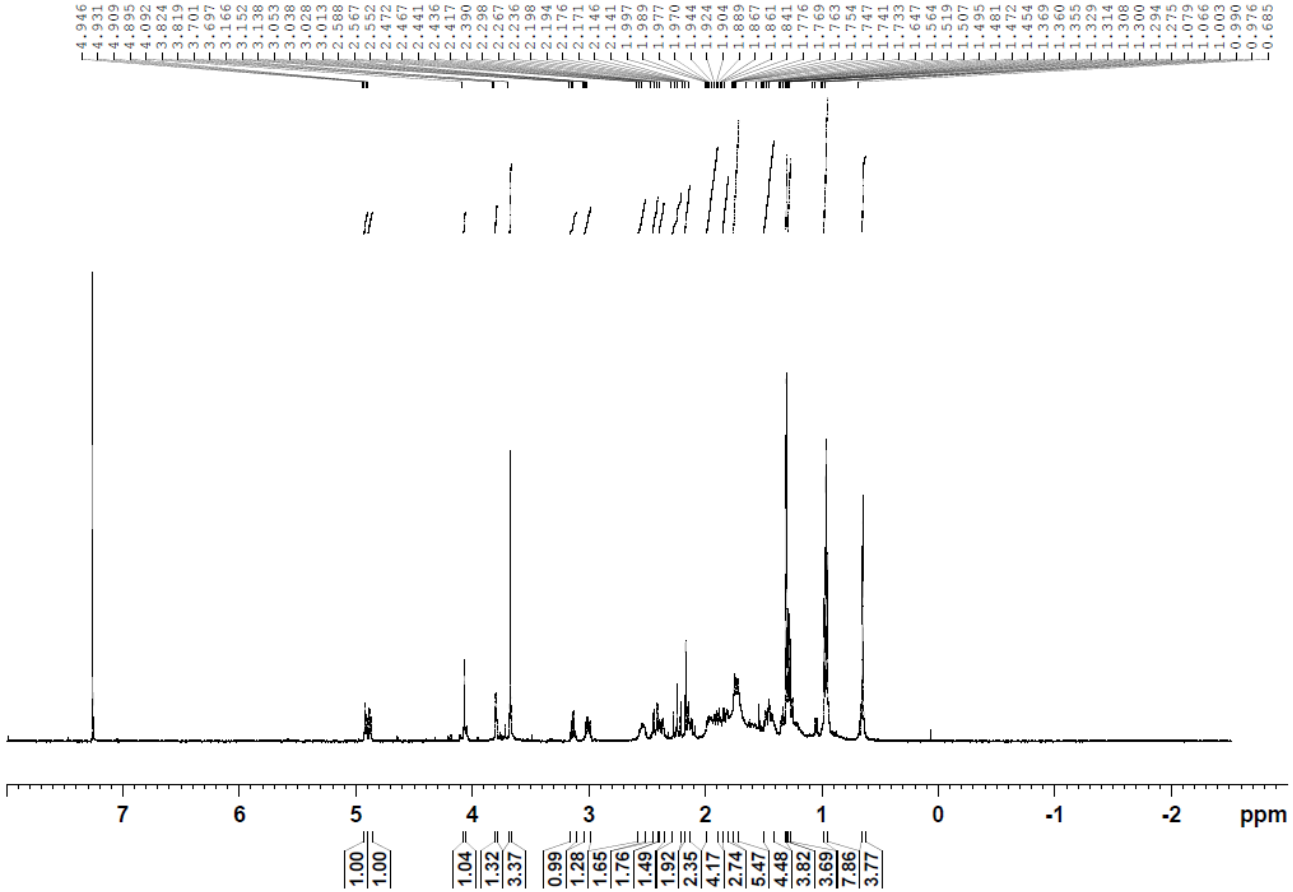

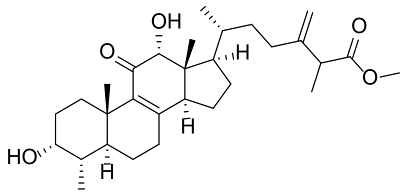

Figure S24 ^{13}C NMR (125 MHz, $CDCl_3$) spectrum of methyl 3β,12β-dihydroxy-11-oxo-4β-methylergosta-8,24(28)-dien-26-oate (methyl antcin M). (28)

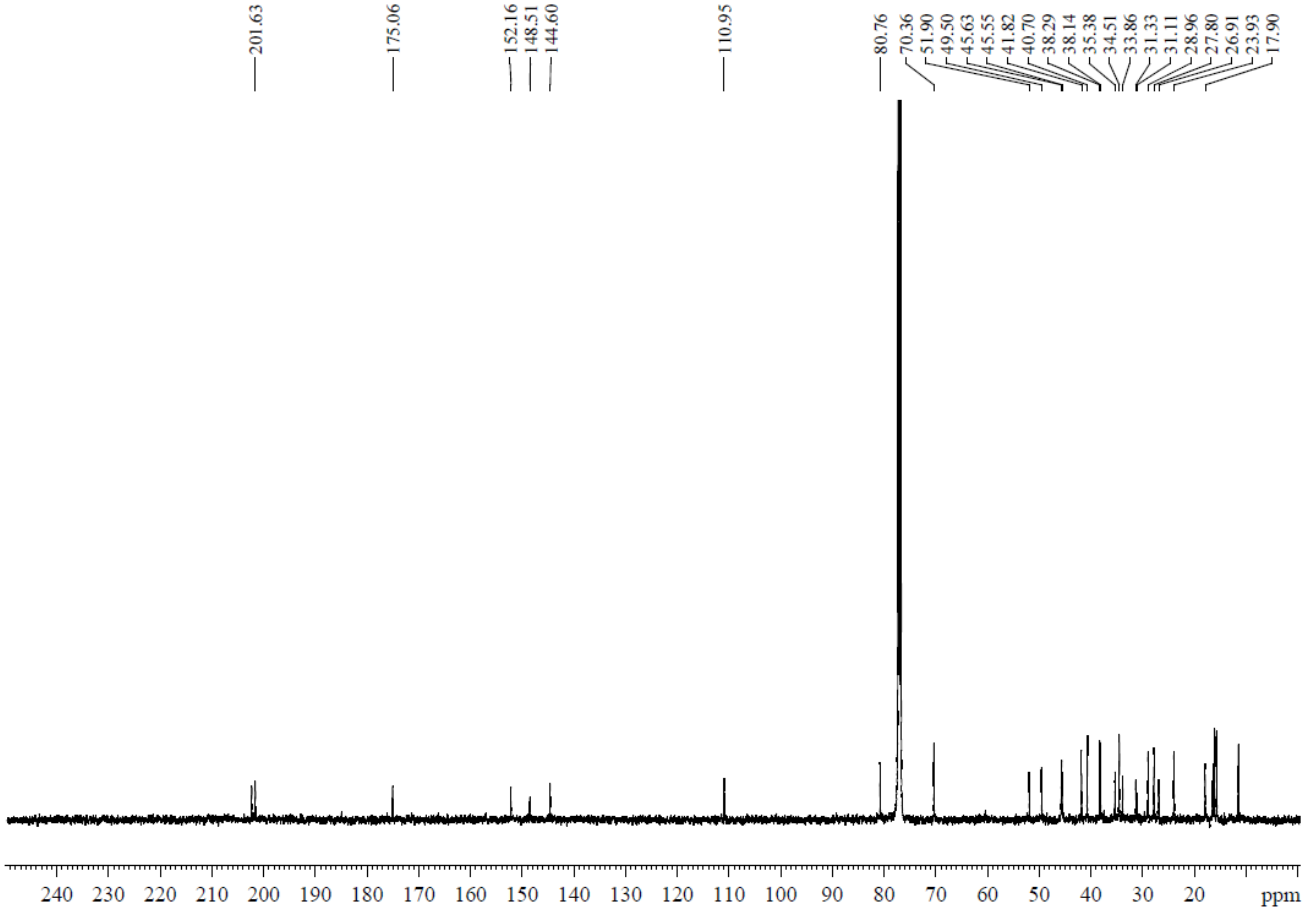

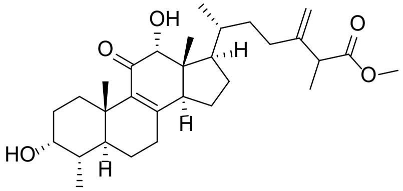

Figure S25 References for known compounds

methyl 3α,7α,12α-trihydroxy-4α-methylergosta-8,24(28)-dien-11-on-26-oate (**6**), [1]

zhankuic acid A methyl ester (**8**), [2]

methyl-7β-hydroxy-3,11-dioxo-4α-methylergosta-8,24(28)-dien-26-oate (**12**), [2]

methyl 3α,12α-dihydroxy 4α-methylergosta-8,24(28)-diene-7,11-dion-26-oate (methyl antcinate H, **16**), [3]

methyl 3α-hydroxy-7,11-dioxo-4α-methylergosta-8,24(28)-dien-26-oate (**20**), [2]

methyl 7β-hydroxy-3,11-dioxo-4α-methylergosta-8,24(28)-dien-26-oate (**22**), [2]

methyl 4α-methylergosta-8,24(28)-diene-3,11-dion-26-oate (**24**), [2]

methyl 3β,12β-dihydroxy-11-oxo-4β-methylergosta-8,24(28)-dien-26-oate (methyl antcin M, **26**) [4]

Reference

[1] C.C. Shen, Y.H. Wang, T.T. Chang, L.C. Lin, M.J. Don, Y.C. Hou, K.T. Liou, S. Chang, W.Y. Wang, H.C. Ko, Y.C. Shen, Anti-inflammatory ergostanes from the basidiomata of *Antrodia salmonea*, Planta Med. 73 (2007) 1208-1213. http://doi.org/10.1055/s-2007-981591

[2] S.J. Wu, W.L. Leu, C.H. Chen, C.H. Chao, D.Y. Shen, H.H. Chan, E.J. Lee, T.S. Wu, Y.H. Wang, Y.C. Shen, K. Qian, K.F. Bastow, K.H. Lee, Camphoratins A−J, Potent Cytotoxic and Anti-inflammatory Triterpenoids from the Fruiting Body of *Taiwanofungus camphoratus*, J. Nat. Prod. 73 (2010) 1756-1762. http://doi.org/10.1021/np1002143

[3] S.W. Yang, Y.C. Shen, C.H. Chen, Steroids and triterpenoids of Antodia cinnamomea—A fungus parasitic on *Cinnamomum micranthum*, Phytochemistry 41 (1996) 1389-1392. http://doi.org/https://doi.org/10.1016/0031-9422(95)00767-9

[4] R. Gautam, S.M. Jachak, Recent developments in anti-inflammatory natural products, Med. Res. Rev. 29 (2009) 767-820. http://doi.org/https://doi.org/10.1002/med.20156
